# Enhancement of binding avidity by bivalent binding enables PrP^Sc^-specific detection by anti-PrP monoclonal antibody 132

**DOI:** 10.1371/journal.pone.0217944

**Published:** 2019-06-06

**Authors:** Akio Suzuki, Takeshi Yamasaki, Rie Hasebe, Motohiro Horiuchi

**Affiliations:** 1 Laboratory of Veterinary Hygiene, Faculty of Veterinary Medicine, Graduate School of Infectious Diseases, Hokkaido University, Kita-ku, Sapporo, Japan; 2 Biomedical Animal Research Laboratory, Institute for Genetic Medicine, Hokkaido University, Kita-ku, Sapporo, Japan; 3 Global Station for Zoonosis Control. Global Institute for Collaborative Research and Education, Hokkaido University, Kita-ku, Sapporo, Japan; Ruhr University Bochum, GERMANY

## Abstract

Anti-prion protein (PrP) monoclonal antibody 132, which recognizes mouse PrP amino acids 119–127, enables us to reliably detect abnormal isoform prion protein (PrP^Sc^) in cells or frozen tissue sections by immunofluorescence assay, although treatment with guanidinium salts is a prerequisite. Despite the benefit of this mAb, the mechanism of PrP^Sc^-specific detection remains unclear. Therefore, to address this mechanism, we analyzed the reactivities of mono- and bivalent mAb 132 to recombinant mouse PrP (rMoPrP) by enzyme-linked immunosorbent assay (ELISA) and surface plasmon resonance (SPR). In ELISA, binding of the monovalent form was significantly weaker than that of the bivalent form, indicating that bivalent binding confers a higher binding stability to mAb 132. Compared with other anti-PrP mAbs tested, the reactivity of bivalent mAb 132 was easily affected by a decrease in antigen concentration. The binding kinetics of mAb 132 assessed by SPR were consistent with the results of ELISA. The dissociation constant of the monovalent form was approximately 260 times higher than that of the bivalent form, suggesting that monovalent binding is less stable than bivalent binding. Furthermore, the amount of mAb 132 that bound to rMoPrP decreased if the antigen density was too low to allow bivalent binding. If two cellular PrP (PrP^C^) are close enough to allow bivalent binding, mAb 132 binds to PrP^C^. These results indicate that weak monovalent binding to monomeric PrP^C^ diminishes PrP^C^ signals to background level, whereas after exposure of the epitope, mAb 132 binds stably to oligomeric PrP^Sc^ in a bivalent manner.

## Introduction

Prion diseases are fatal neurodegenerative diseases in animals and humans, including bovine spongiform encephalopathy, scrapie in sheep and goats, and Creutzfeldt-Jakob disease in humans [1]. The causative agents of prion diseases, prions, are mainly composed of an abnormal isoform of prion protein (PrP^Sc^), which is generated from a host-encoded cellular isoform of prion protein (PrP^C^) by certain post-translational modifications including conformational transformation. After prion infection, production of PrP^Sc^ commences in the central nervous system during a long latency period, and eventually neuronal cell death is caused by production of PrP^Sc^ in neurons itself or an alteration of the neural niche as a response to PrP^Sc^ formation and accumulation. A critical question in elucidating the pathogenesis of prion diseases is where and how PrP^Sc^ accumulates in the central nervous system as disease progress.

PrP^Sc^ is distinguished from PrP^C^ by several biochemical and biophysical properties such as a high β structure content, resistance to protease digestion, and insolubility to nonionic detergent [2]. Hydrated autoclaving is commonly used to detect PrP^Sc^ from tissue sections [3]. Removal of PrP^C^ by proteinase K (PK) also enables us to detect PrP^Sc^ by immunocytochemistry [4]. However, these methods involve harsh treatments that affect tissue or cell architecture. In addition, it has been reported that PrP^Sc^ comprises PK-resistant PrP^Sc^ (PrP^Sc^-res) and PK-sensitive PrP^Sc^ (PrP^Sc^-sen) [5,6] and that PrP^Sc^-sen exhibits higher infectivity and higher conversion-inducing activity than PrP^Sc^-res [7]. Thus, immunocytochemical and/or immunohistochemical methods that can distinguish PrP^Sc^ from PrP^C^ without severe damage to cell or tissue structures and that can detect PrP^Sc^-sen are required for detailed analyses of the mechanism of prion propagation and the pathobiology of prion diseases. Several PrP^Sc^-specific mAbs have been produced [8–13]; however, their ability to specifically detect PrP^Sc^ in immunocytochemical analysis is largely unclear.

Despite such technical limitations, PrP^Sc^-specific staining using pan-PrP antibodies in immunofluorescence assay (IFA) after treatment of fixed cells with a chaotropic agent such as guanidinium (Gdn) salt [14] has partly revealed the localization of PrP^Sc^ in prion-infected cells [15–17]. However, since most pan-PrP mAbs react with both PrP isoforms, manipulation of the detector gain or exposure time is required to set a threshold level just above the PrP^C^ signals. Recently, we reported that the mAb 132 recognizing mouse PrP amino acids (aa) 119–127, is useful for reliable PrP^Sc^-specific staining in cells or frozen tissue sections by IFA without PK treatment [18–20] and flow cytometry [21], even though this mAb is classified as a group of pan-PrP antibodies that is incapable of distinguishing PrP^Sc^ from PrP^C^. We also reported that mAb 132 detects both PrP^Sc^-sen and PrP^Sc^-res if not all [22]. Although brief pretreatment of cells or tissue sections with guanidinium salt is still required for PrP^Sc^-specific staining with mAb 132, this mAb shows little reactivity with native PrP^C^ in cells and tissues so that manipulation of conditions for acquiring fluorescence images can be minimized.

Despite the benefit of mAb 132 in PrP^Sc^-specific staining, the mechanism of this PrP^Sc^-specific detection remains unclear. In the present study, we analyzed the reactivity and binding kinetics of mAb 132 and its derivatives to understand the mechanism of PrP^Sc^-specific detection by mAb 132.

## Materials and methods

### Hybridomas culture and RNA extraction

Hybridomas producing mAbs 132 (epitope: mouse PrP aa 119–127), 31C6 (epitope: mouse PrP aa 143–149), and 44B1 (epitope: discontinuous epitope comprised of mouse PrP aa 155–231) were cultured as described elsewhere [23]. Total RNA was extracted from hybridomas using TRIzol reagent (Life Technologies, USA) according to the manufacturer’s instructions.

### Cloning of immunoglobulin genes and construction of expression plasmids

Methods used to clone heavy and light chains of immunoglobulin genes and construct expression plasmids for recombinant antibody fragments are given in the S1 Text. Accession numbers of the heavy and light chain genes of each mAb are as follow: mAb 132 light (LC028385) and heavy (LC028384) chains, mAb 31C6 light (LC026057) and heavy (LC026056) chains, and mAb 44B1 light (LC037231) and heavy (LC037230) chains.

### Expression of recombinant antibodies

HEK293T cells were split in a ratio of 1:10 into 6 cm dishes 36 h before transfection. Plasmids for expression of recombinant antibody fragments (4 μg) were transfected using Lipofectamine 2000 reagent (Invitrogen, USA) according to the manufacturer’s instructions. Twenty-four hours after transfection, the medium was replaced with DMEM containing 10% fetal bovine serum (FBS) (Gibco, USA) and 1% Penicillin Streptomycin (Gibco) and the cells were incubated for 24 h. Then, the cells were fed with fresh Opti-MEM and incubated for further 48 h and 96 h, for the expression plasmids containing an internal ribosomal entry site (IRES) [24] and a self-cleavage 2A peptide from foot-and-mouth disease virus (F2A) peptide [25], respectively.

Transfection of the expression plasmids containing IRES and F2A into 293F cells (Invitrogen) was performed according to the manufacturer’s instructions with minor modifications. The 293F cells passaged at least five times, were split at 0.7 × 10^6^/ml into 250 mL spinner flasks (Corning, USA) using 80–100 ml FreeStyle 293 Expression Medium (Gibco) containing 0.5% Antibiotic/Antimycotic (Gibco). After incubation for 24 h, plasmids mixed with 293Fection Transfection reagent (Invitrogen) in Opti-Pro SFM (Gibco) were added into the 293F cell suspension at 1.0 × 10^6^/ml with FreeStyle 293 Expression Medium. The cells were cultured for 4 days at 37°C with 8% CO_2_.

### Purification of recombinant antibodies

The culture supernatants from 293F cells were dialyzed twice against phosphate-buffered saline (PBS, pH 7.2) using Spectra/Pore 7 (Funakoshi, Japan) at 4°C. After removal of precipitates by centrifugation at 2,300 × *g* for 5 min at 4°C, the supernatant was loaded onto a Ni^2+^-immobilized 5 ml HiTrap Chelating HP column (GE Healthcare, UK) by circulating the supernatant for 6 h. After washing the column with 20 ml PBS, 1 M imidazole in PBS was applied to elute bound materials and the eluates were manually collected as 1 ml fractions. Fractions containing recombinant (r)Fab-132, rFab-132-EGFP, and rIgG-132-EGFP were further loaded onto a HiLoad 16/600 Superdex 200 pg column (GE Healthcare) and separated using the ÄKTAexplore 10S system (GE Healthcare) at a flow rate of 1.5 ml/min. After discarding the first 20% column volume as a void fraction, the isocratic eluates were collected in 1.5 ml fractions. In the case of rIgG-132, Protein G Sepharose 4 Fast Flow (GE Healthcare) was used for purification.

### Immunoblotting

SDS-PAGE and western transfer under non-reducing condition were carried out as described previously [23] without using 2-mercaptoethanol or an antioxidant. After blocking of Immobilon-P transfer membrane (Millipore, USA), Fab regions were directly probed with 1:5,000 diluted Anti-Mouse IgG (Fab specific)- Peroxidase antibody produced in goat (Sigma, USA). Antigen-antibody complexes were visualized with ECL Western Blotting Detection Reagents (GE Healthcare) using an LAS-3000 chemiluminescence image analyzer (Fujifilm, Japan).

### ELISA

ELISA using rMoPrP as antigen was carried out as described elsewhere [23]. The rMoPrP pretreated with 6 M GdnHCl (2 ng/μl) was adsorbed to the wells (100 ng/well) of a U96 Maxisorp Nunc-immuno plate (Nunc, Denmark) overnight at room temperature (r.t.). After blocking with 5% FBS in PBS containing 0.05% Tween 20 (PBST), the wells were incubated with primary antibodies for 1 h at r.t. After washing with PBST, wells were incubated with 100 μl of 1:5,000 diluted Anti-Mouse IgG (Fab specific)- Peroxidase antibody produced in goat in PBST containing 0.5% FBS. Finally, antigen-antibody complexes were detected by adding 100 μl of 3, 3’, 5, 5’-tetramethylbenzidine (Sigma) colorimetric substrate, and the absorbance at 450 nm was measured with an Acent micro-plate reader (Labsystems).

### PrP^Sc^-specific immunofluorescence staining

PrP^Sc^-specific immunofluorescence staining was performed as described elsewhere with minor modification [20,26]. Briefly, cells in eight-well chamber were fixed with 4% paraformaldehyde (PFA), and remaining PFA was quenched with 100 mM glycine in PBS. Cells were then permeabilized with 0.1% Triton X-100 in PBS, and treated with 5 M GdnSCN for 15 min at r.t. After blocking with 5% FBS in PBS (FBS-PBS) for 30 min at r.t., cells were incubated with recombinant mAb fragments overnight at 4°C. Cell nuclei were stained with 4’, 6’-diamidino-2-phenylindole, dilactate (DAPI).

The immunofluorescence staining of frozen sections was performed as described elsewhere [18,21] with minor modifications. Briefly, the frozen brain sections were fixed and permeabilized as described above. Then, the sections were treated with 2.5 M GdnSCN for 15 min at r.t. The sections were blocked with 5% FBS in PBS (FBS-PBS) for 1 h at r.t. and were incubated with anti-PrP mAbs or anti-feline panleukopenia virus mAb P2-284 [27] used as a negative control at 1 μg/ml in 1% FBS-PBS overnight at 4°C. The bound antibodies were probed with 0.4 μg/ml Alexa Fluor 488 F(ab’)_2_ fragment of goat anti-mouse IgG (H+L) (Invitrogen) in 1% PBS-PBS for 1 h at r.t. Cell nuclei were stained with DAPI, and the sections were mounted with ProLong Glass antifade mountant (Invitrogen). The fluorescent images were acquired using LSM700 and analyzed with ZEN software (Zeiss) as described elsewhere [19,26].

### SPR

The affinity of antibody binding was determined from SPR sensorgrams measured in multi- and single-cycle kinetics analysis using Biacore X100 (GE Healthcare). Multi-cycle kinetics analysis was performed as follows: mAbs 31C6 and 132 were immobilized on the surface of a CM5 sensorchip as ligands (GE Healthcare) at approximately 1,000 RU according to the manufacturer’s instructions. The real-time association responses of injected rMoPrP as an analyte to antibodies were measured for 180 sec. After measuring the association phase, the dissociation responses were measured while the surface of the sensorchip was washed with HBS-EP+ buffer (GE Healthcare) for 600 sec. The sensorchip was regenerated with 10 mM NaOH according to the manufacturer’s instructions.

Single-cycle kinetics analysis using rMoPrP as a ligand was performed as follows: 1,706.7 and 140.9 RU of rMoPrP were immobilized on a CM5 sensorchip using 1 μM and 100 nM rMoPrP, respectively. The association responses of antibodies to rMoPrP were measured for 120 sec, and then the dissociation of antibodies were measured for 3,600 sec for IgG-31C6 and IgG-132, and 600 sec for rFab-31C6 and rFab-132, respectively. For repeated measurements, the sensorchip was regenerated once (80 sec for rFab-31C6 and IgG-31C6) or twice (80 and 60 sec for rFab-132 and IgG-132, respectively) with 10 mM NaOH.

The binding responses of IgG-132 and IgG-31C6 captured on the sensorchip to rMoPrP were measured as follows. Anti-mouse IgG antibody in the Mouse Antibody Capture Kit (GE Healthcare) was immobilized on a CM5 sensorchip according to the manufacturer’s instructions. Then 10 nM of IgG was applied until the resonance reached approximately 500 RU. The association and dissociation responses for injected rMoPrP were measured for 120 and 600 sec, respectively. Data processing and determination of association rate constant (*k*_*a*_), dissociation rate constant (*k*_*d*_), and dissociation constant (*K*_*D*_) by fitting were performed using BIA evaluation software (GE Healthcare).

## Results

### Production of recombinant mAb132

**Fig 1:**
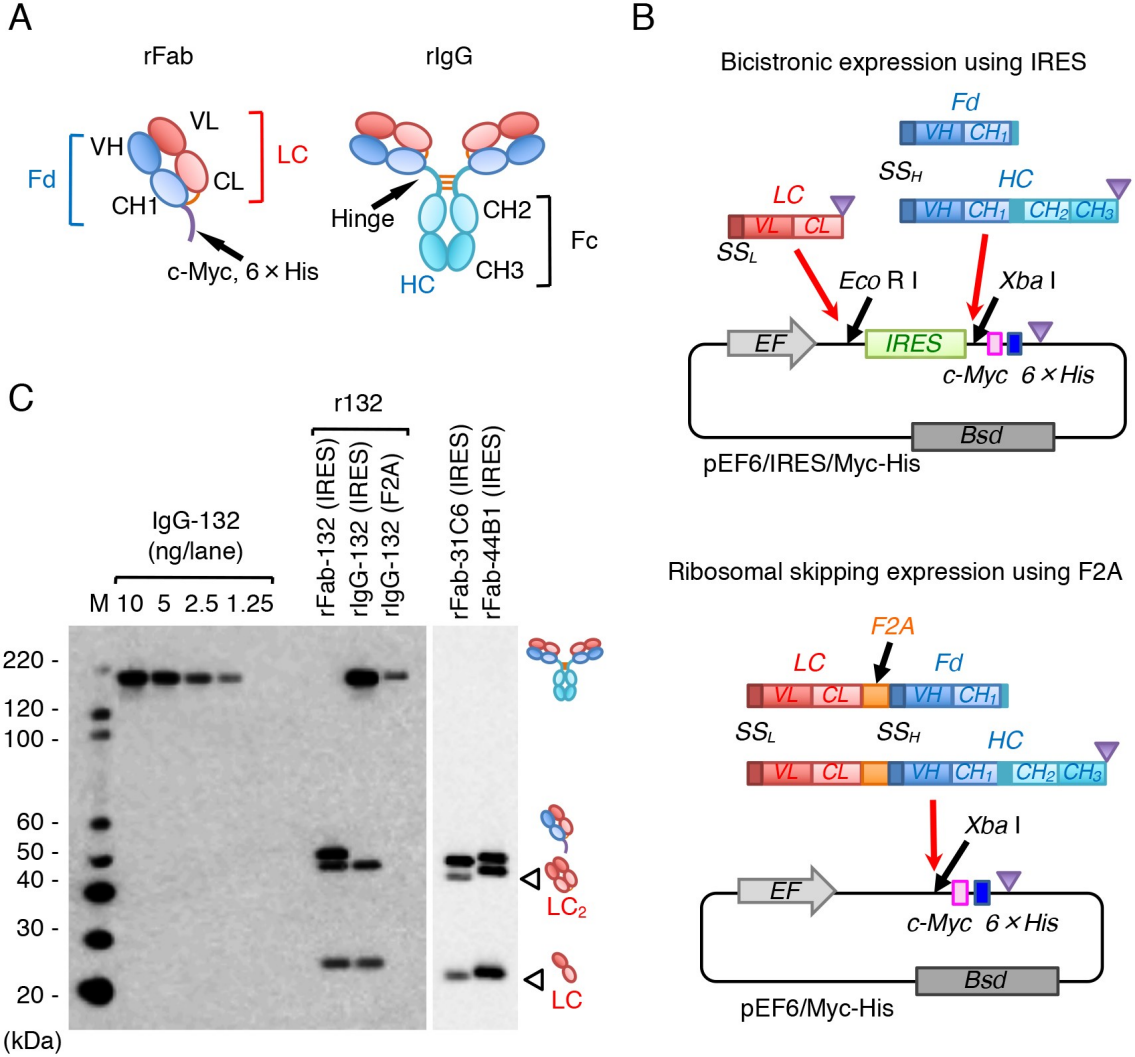
Production of recombinant antibody fragments. (A) Molecular design of mono- (rFab) and bivalent forms (rIgG). The light chain and full length (HC) or VH-CH1 region (Fd) of the heavy chain are depicted with reddish and bluish colors, respectively. Intermolecular disulfide bonds are drawn in orange lines, whereas the C-terminal epitope tail consisting of a c-Myc epitope (c-Myc) and a hexa-histidine tag (6 × His) is drawn in purple. Fc, fragment crystallization; CH1-3, constant domains 1–3 of heavy chain; CL, constant domain of light chain, VH, variable region of heavy chain; VL variable region of light chain. (B) Schematic illustration of two expression systems. DNA fragments for an internal ribosome entry site (IRES) and self-cleavage 2A peptide from foot-and-mouth virus (F2A) are shown in green and orange, respectively. For producing antibody fragments into culture supernatants, heavy and light chain genes possess their own signal sequences (SS_H_ and SS_L_, respectively) at the 5'-terminus. The gene encoding the Fd region was amplified without a stop codon (purple triangles) to add the epitope tags c-Myc and 6 × His at the 3'-terminus (S1B Fig and S2 Table). EF, a promoter of the human elongation factor gene; Bsd, blasticidin-resistant gene. (C) Analysis of molecular assembly of antibody fragments. Immunoblot analysis was carried out using authentic mAb 132 (IgG-132, 1.25–10.0 ng/lane) and supernatants of HEK293T cells transfected with expression plasmids containing IRES (rFab, rIgG) or F2A (rIgG) under non-reducing condition. Blot was probed with an HRP-conjugated anti-mouse IgG (Fab specific) (Sigma). Corresponding pictures on the right indicate the bands of IgG, rIgG, or rFab, whereas arrowheads indicate the bands of free and dimeric LC (LC_2_). Molecular mass markers on the left are in kDa.

MAb 132 hardly reacts with native PrP^C^ on the cell surface [28] but reacts with denatured PrP^Sc^ and PrP^C^ in immunoblot analysis and with recombinant PrP in enzyme-linked immunosorbent assay (ELISA) [23], suggesting that this mAb is classified as a pan-PrP mAb. To examine the mechanism of the PrP^Sc^-specific detection by mAb 132 in IFA, we cloned immunoglobulin heavy and light chain genes and constructed expression plasmids for mono- and bivalent forms of recombinant antibodies (Fig 1A) and their derivatives (S1 Fig).

We used two expression systems such that two peptide chains could be co-expressed from a single plasmid (Fig 1B); one system was bicistronic expression using an internal ribosomal entry site (IRES) [24] and the other was C-terminal cleavage by ribosomal skipping using a self-cleavage 2A peptide from foot-and-mouth disease virus (F2A) peptide [25]. Regarding the F2A peptide expression plasmid, we could construct expression plasmids using F2A for rIgG of mAb 132 (rIgG-132) but not for rFab of mAbs 132, 31C6, or 44B1 because these constructs could not be subcloned without an irregular single base insertion in the F2A sequence. The expression and assembly of rIgG-132 and rFabs in the culture supernatants of HEK293T cells transfected with each expression plasmid were assessed by immunoblotting (Fig 1C). Expression plasmids with F2A produced a lesser amount of rIgG-132 than those with IRES. However, bicistronic expression using IRES produced a considerable amount of free light chain and light chain dimers (Fig 1C, LC, and LC_2_, respectively), which were not observed in the supernatant of cells transfected with expression plasmids with F2A. The production of free light chains and light chain dimers was also observed in rFabs (rFab-132, rFab-31C6 and rFab-44B1) using IRES (Fig 1C). These results suggest that expression plasmids with F2A peptide are preferable for producing recombinant antibodies because of traces of unwanted byproducts such as free and dimeric light chains.

### Reactivity of mono- and bivalent forms of recombinant mAb 132 fragments to recombinant mouse PrP

**Fig 2:**
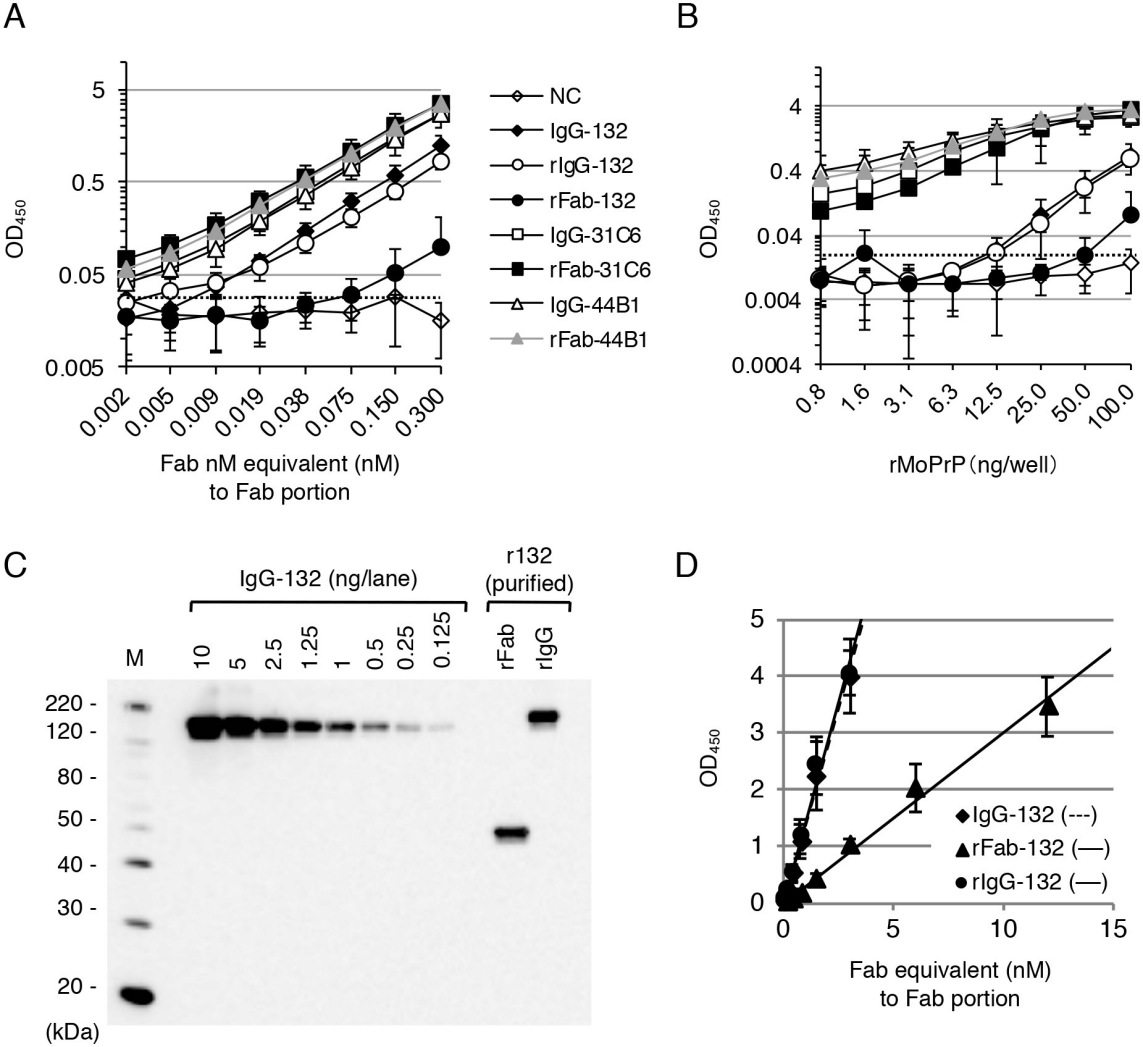
Reactivity of recombinant antibodies in ELISA. (A) Reactivity of authentic and recombinant antibodies. Twofold serially diluted antibodies (0.002–0.3 nM Fab equivalent) were added to wells coated with 100 μg/well rMoPrP. Purified IgG was used for authentic mAbs, whereas the supernatant of HEK293T cells transfected with each expression plasmid was used for recombinant antibodies after estimation of concentrations equivalent to the Fab portion by immunoblot analysis using serially diluted purified IgG as a standard. Reactivities of authentic IgG and rIgG of mAbs 132, 31C6, and 44B1 as well as their rFab fragments are shown. Mean absorbances at 450 nm and SD (three independent experiments) are plotted. The supernatant of HEK293T cells transfected without any plasmids was used as a negative control (NC). Dotted line indicates a cutoff value calculated by the average plus 3 × SD. (B) Antigen concentration-dependent reactivity of authentic and recombinant antibodies. Twofold serially diluted rMoPrP (0.8–100 ng/well) was adsorbed to wells. For detection of rMoPrP, 0.3 nM antibodies equivalent to the Fab portion were used. (C) Quantification of purified rFab-132 and rIgG-132. Concentrations of recombinant antibodies were estimated by comparing 2-fold serially diluted authentic IgG-132 (0.125–10 ng). Blot was probed with an HRP-conjugated anti-mouse IgG (Fab specific) (Sigma), and signal intensities were quantified using Image Gauge software (Fujifilm). (D) Difference in reactivity of monovalent (rFab-132) and bivalent (IgG-132 and rIgG-132) forms of mAb 132. ELISA was carried out using 100 ng/well rMoPrP as an antigen and twofold serially diluted purified rFab-132 (0.09–12 nM), IgG-132, and rIgG-132 (0.02–3 nM) as primary antibodies.

Next, we examined the reactivity of the mono- and bivalent form of anti-PrP mAb 132 to recombinant mouse PrP (rMoPrP) with rFab and IgG, respectively, using ELISA (Fig 2). Supernatants of HEK293T cells transfected with each expression plasmid were used as sources of recombinant antibodies after estimation of concentrations equivalent to Fab portion by immunoblot analysis. The reactivities of rFab-31C6 and rFab-44B1, recombinant Fab fragments of pan-PrP mAbs 31C6 and 44B1, respectively, were comparable to those of the corresponding authentic IgGs. In contrast, the reactivity of rFab-132 to rMoPrP was much weaker than that of authentic IgG (IgG-132) and recombinant mAb 132 (rIgG-132). The rFab-132 reacted very weakly even at the highest concentration used (0.3 nM), whereas rIgG-132 and its authentic IgG reacted positively even at concentration the 30-fold weaker (0.009 nM) (Fig 2A). Fig 2B shows the antigen concentration-dependent reactivity of these mAbs and rFabs. Again, rFab-31C6 and rFab-44B1 showed similar reactivity to the corresponding IgGs; however, compared with the authentic IgG-132 and rIgG-132, rFab-132 could not bind efficiently to rMoPrP. To confirm the reactivity of the mono- and d-valent forms of mAb 132, rIgG-132 and rFab-132 were purified from the supernatants of spinner-cultured HEK293F cells transfected with the corresponding expression plasmids using Protein G and Ni^2+^ affinity chromatography, respectively. Immunoblot analysis of the purified antibody fragments demonstrated efficient removal of the light chain products (Fig 2C). Both IgG-132 and rIgG-132 gave a result of 3 OD_450_ at around 2 nM, whereas approximately 10 nM of rFab-132 was required to obtain the same level of reaction (Fig 2D). In this experiment, IgG concentration was estimated according to the Fab portion. Thus, at the same concentration, the number of IgG molecules was half of that of Fab fragments. Therefore, the reactivity of rFab-132 was estimated as roughly one-tenth of that of the IgG form.

### Production of recombinant mAb 132-EGFP fusion proteins

**Fig 3:**
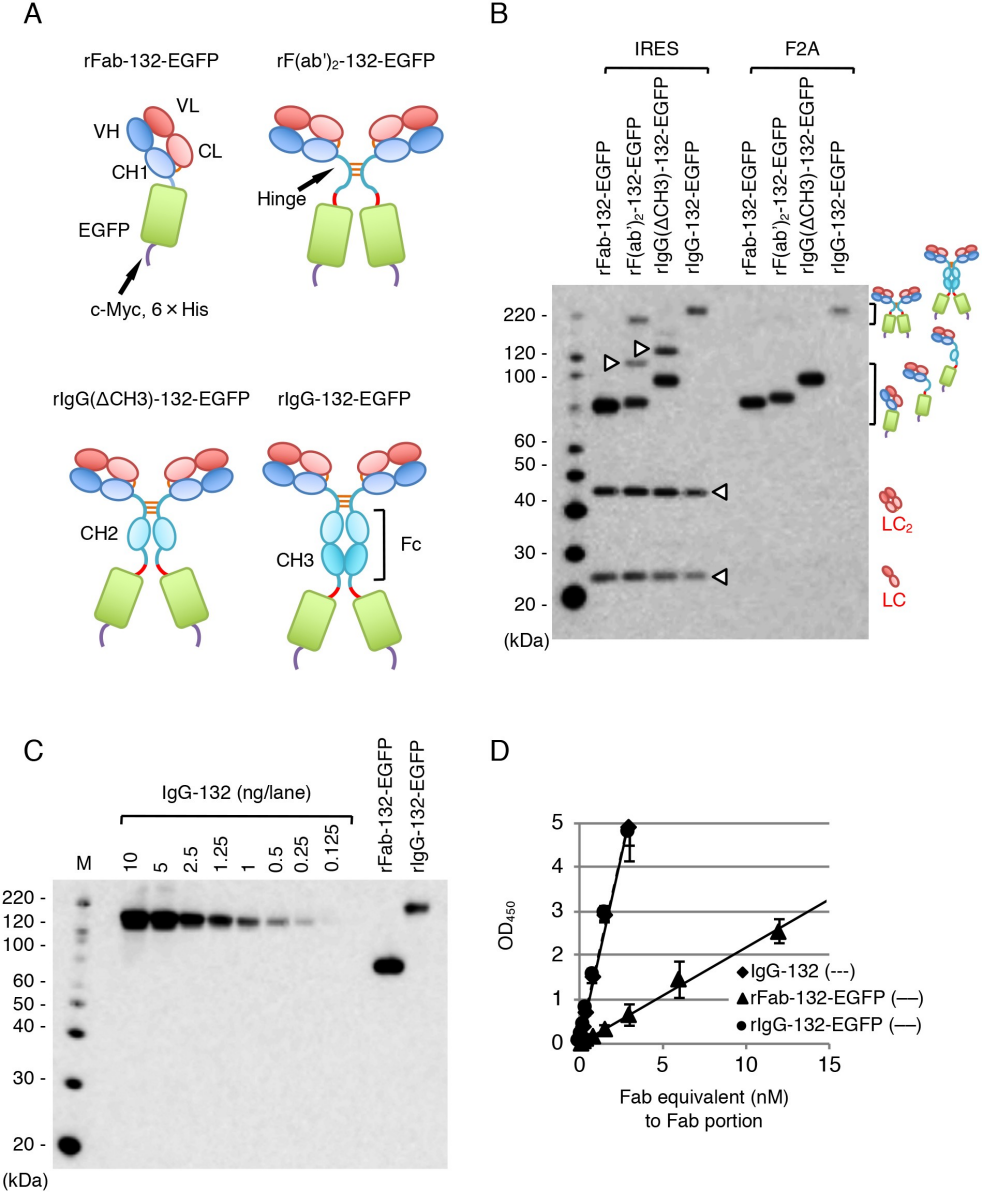
Reactivity of mAb 132-EGFP fusion proteins. (A) Molecular design of mAb 132-EGFP fusion proteins. The light and heavy chains are depicted as in Fig 1A, and the spacer between heavy chains and EGFP is indicated with red lines. The epitope tag consisting of c-Myc epitope and a 6 × His (purple lines) was added at the C-terminus of EGFP. (B) Analysis of the molecular assembly of mAb 132-EGFP fusion proteins. Expression plasmids were introduced into HEK293T cells and mAb 132-EGFP fusion proteins were detected with a HRP-conjugated anti-mouse IgG (Fab specific) (Sigma). Corresponding illustrations on the right indicate the products of rFab-EGFP, rF(ab’)_2_-132-EGFP, rIgG(ΔCH3)-132-EGFP and rIgG-132-EGFP, whereas arrowheads indicate possible assembly intermediates, free light chain and free light chain dimer (LC_2_). The square brackets indicate the molecular weight range of mono- and bivalent fusion proteins. (C) Quantification of purified rFab-132-EGPG and rIgG-132-EGFP. Concentrations of fusion proteins were estimated by comparing 2-fold serially diluted authentic IgG-132 (0.125–10 ng). Blot was probed with an HRP-conjugated anti-mouse IgG (Fab specific), and signal intensities were quantified. (D) Difference in reactivity of monovalent (rFab-132-EGFP) and bivalent (rIgG-132-EGFP) form of mAb 132-EGFP fusion proteins. ELISA was carried out using 100 ng/well of rMoPrP as an antigen and 2-fold serially diluted purified rFab-132-EGFP (0.09–12 nM), rIgG-132-EGFP, and IgG-132 (0.02–3 nM) as primary antibodies.

MAb 132 fused with EGFP could be useful for a direct detection of PrP^Sc^ in multiple immunofluorescence staining. Thus, we attempted to produce four EGFP fusion proteins, rFab-132-EGFP, rF(ab’)_2_-132-EGFP, rIgG(ΔCH3)-132-EGFP lacking the CH3 domain of the constant region, and rIgG-132-EGFP (Fig 3A). The EGFP gene was fused to the 3' ends of gene fragments encoding various heavy chain genes of mAb 132 via a DNA fragment encoding a spacer peptide GGGGSGGGGSGGGGS for the bivalent form of recombinant mAb 132 derivatives (Fig 3A) because the spacer would be useful in avoiding an interaction that affects proper folding of the heavy chain Fd region and EGFP [29,30]. The recombinant mAb 132-EGFP fusion proteins were expressed in HEK293T cells by a single expression plasmid with either IRES or F2A peptide.

Fig 3B shows the expression and assembly of recombinant mAb 132-EGFP fusion proteins in the culture supernatants of HEK293T cells. Similar to the expression of rFab-132 and rIgG-132 in Fig 1C, bicistronic expression using IRES produced free light chain, light chain dimers, and assembly intermediates (Fig 3B, indicated with arrowheads), which were not observed with expression plasmids with F2A. Bivalent antibodies were produced from the expression plasmid containing a cDNA fragment encoding the CH3 domain of the Fc region (rIgG-132-EGFP) but not from those lacking cDNA for the CH3 domain [rF(ab’)_2_-132-EGFP and rIgG(ΔCH3)-132-EGFP] (Fig 3B). These results indicate that the CH3 domain is required for efficient formation of hetero-tetrameric IgG molecules consisting of two light chains and two heavy chains, as reported previously [31].

The rFab-132-EGFP and rIgG-132-EGFP were purified by Ni^2+^ affinity chromatography from supernatants of HEK293F cells transfected with corresponding expression plasmids with F2A (Fig 3C), and their reactivity to rMoPrP was analyzed. Similar to the difference in the reactivity of rFab-132 and rIgG-132 to rMoPrP (Fig 2D), the reactivity of the monovalent form of EGFP fusion protein to rMoPrP was much weaker than that of the bivalent form (Fig 3D).

### Effect of valencies of mAb 132 on PrP^Sc^-specific detection

**Fig 4:**
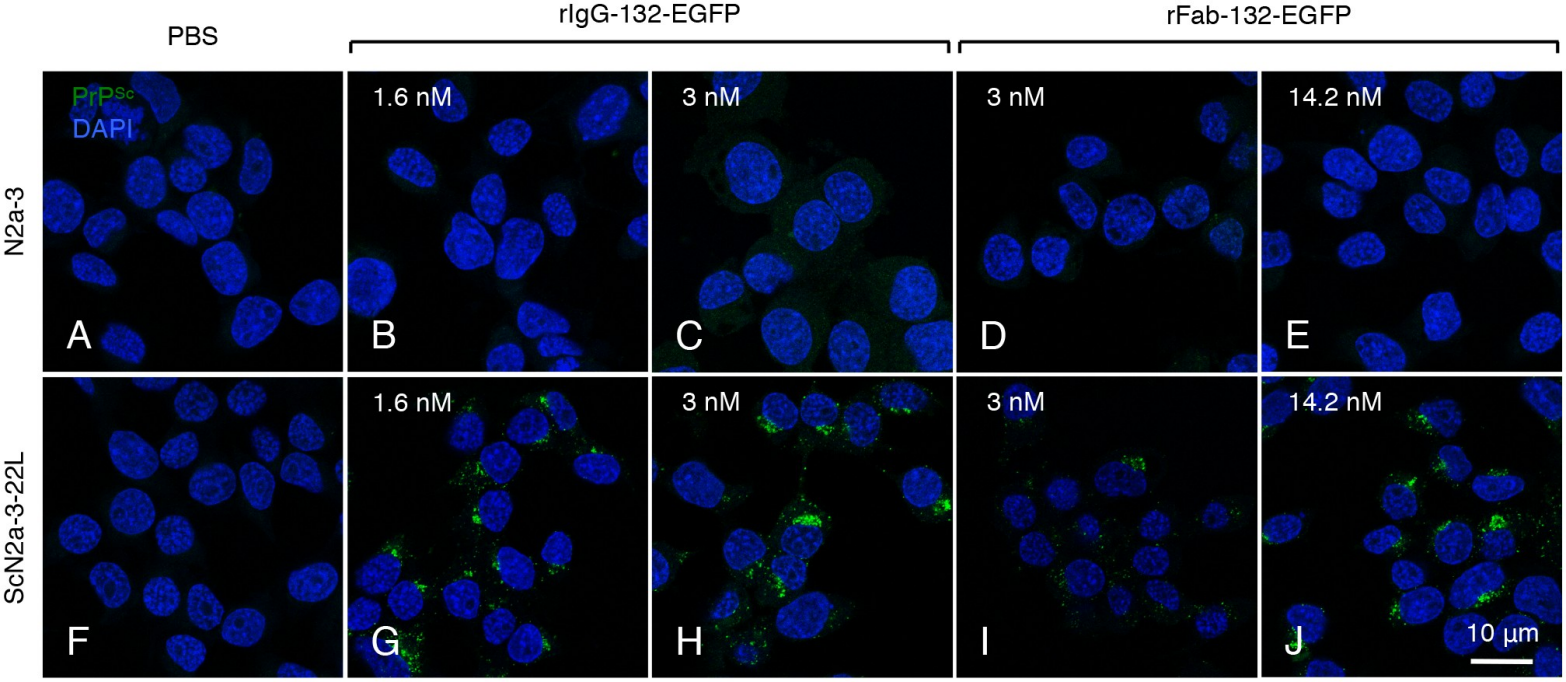
PrP^Sc^-specific detection by direct immunofluorescence staining. Prion-uninfected N2a-3 cells (A–E) and prion 22L strain-infected Na-3 cells (ScN2a-3-22L) (F–J) cells were directly stained using rIgG-132-EGFP (B, C, G, and H) and rFab-132-EGFP (D, E, I, and J) at the indicated concentrations equivalent to the Fab portions. The leftmost images (A, F) show negative controls for EGFP fusion proteins. Cell nuclei were stained with DAPI (blue).

Next we examined the reactivity of the mono- and bivalent forms of recombinant mAb 132 in PrP^Sc^-specific immunofluorescence staining using N2a-3 cells persistently infected with the 22L prion strain (ScN2a-3-22L) (Fig 4). The rIgG-132-EGFP showed typical perinuclear PrP^Sc^ stains as described previously [19,20], even at the lowest concentration used (Fig 4G). The rFab-132-EGFP reacted very weakly to the perinuclear PrP^Sc^ at 3 nM equivalent to the Fab portion (Fig 4I), whereas intense perinuclear stains were observed if the same concentration of rIgG-132-EGFP was used (Fig 4H). If cells were stained with a higher concentration of rFab-132-EGFP (at 14.2 nM equivalent to the Fab portion), at which OD_450_ values obtained with rFab-132-EGFP in ELISA were almost comparable to those obtained with rIgG-132-EGFP at 1.6 nM equivalent to the Fab portion (Fig 3D), peri-nuclear PrP^Sc^ stains were apparent in ScN2a-3-22L cells (Fig 4J). These results suggest that bivalent binding of mAb 132 is required for efficient PrP^Sc^-specific staining.

### Analysis of the binding characteristics of mAb 132 by surface plasmon resonance

**Fig 5:**
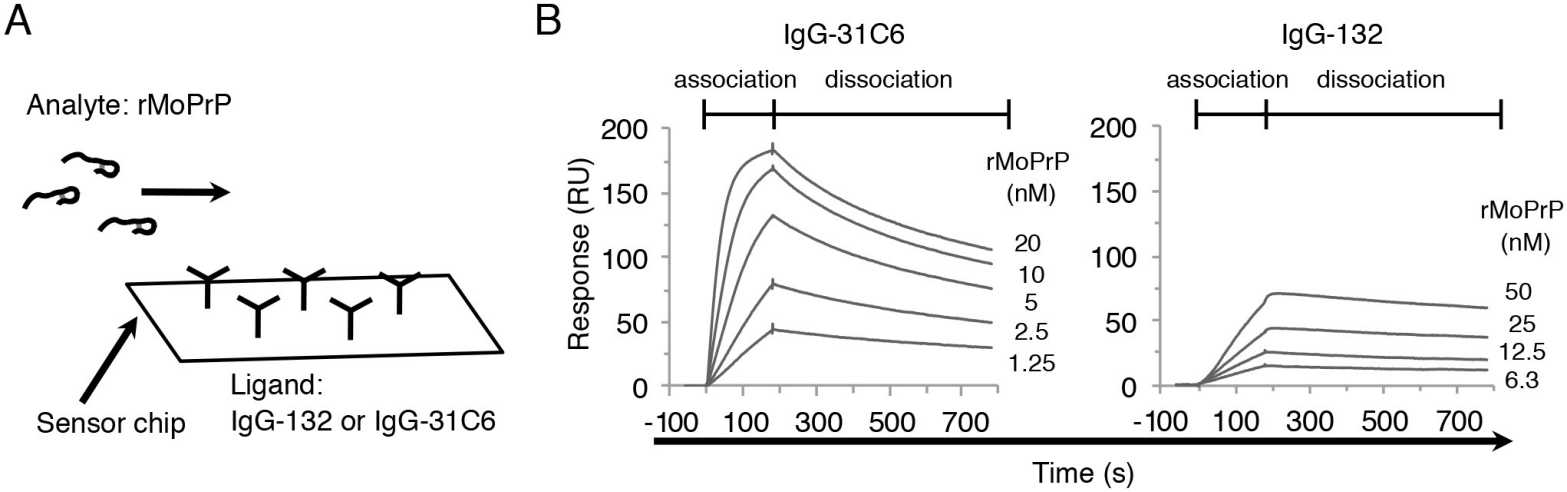
Binding affinity of authentic mAbs. (A) Relationship between ligand and analyte. IgG-132 or IgG-31C6 was directly immobilized at approximately 1,000 RU on the surface of a CM5 sensorchip as a ligand. The rMoPrP was passed over the surface of the chip as an analyte. (B) Multi-cycle kinetics analysis. SPR sensorgrams for the binding of anti-PrP mAb to rMoPrP were obtained using 2-fold serial dilutions of rMoPrP (1.25–20 nM for IgG-31C6 and 6.3–50 nM for IgG-132).

To analyze the effect of antibody valency and antigen density on the reactivity of mAb 132 in more detail, we measured the binding kinetics of mono- and bivalent mAb 132 to rMoPrP using surface plasmon resonance (SPR). We first measured binding responses using IgG (mAbs 31C6 and 132) immobilized on the surface of a sensorchip as a ligand and rMoPrP as an analyte (Fig 5A). In the association phase, the slopes of the sensorgram for IgG-132 were gentler than those for IgG-31C6, indicating that the affinity of IgG-132 to rMoPrP is lower than that of IgG-31C6. In contrast, the decrease of response units (RU) in IgG-132 was slower than that in IgG-31C6, indicating that the binding stability between IgG-132 and rMoPrP is higher than that between IgG-31C6 and rMoPrP. Moreover, IgG-132 required a 20-fold higher concentration of rMoPrP to obtain RU levels comparable to IgG-31C6 (Fig 5B, e.g., compare 1.25 nM rMoPrP for IgG-31C6 with 25 nM rMoPrP for IgG-132), which was consistent with the results that a larger amount of rMoPrP per well was required to obtain efficient binding of mAb 132 in ELISA (Fig 2B).

**Fig 6:**
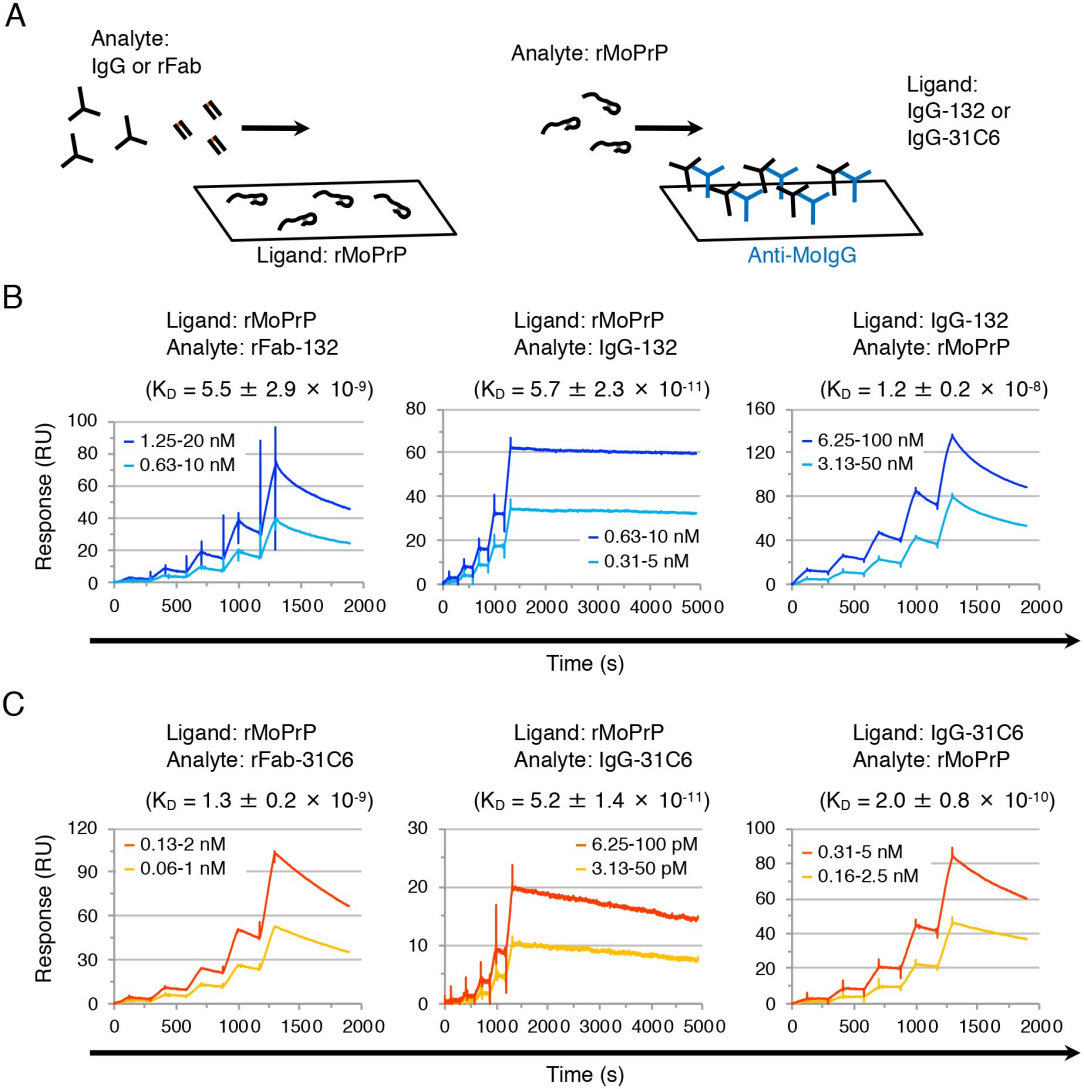
Binding affinity of mono- and bivalent authentic and recombinant antibody fragments. (A) Schematic illustrations for analytes and ligands. The SPR sensorgrams were measured using two different binding types: mono- or bivalent antibody fragments were injected to the sensorchip with immobilized rMoPrP (left), or rMoPrP was injected to the sensorchip with each IgG (at approximately 500 RU) captured by anti-MoIgG (blue) immobilized on the surface (right). (B) Representative sensorgrams for the binding of authentic and recombinant antibody fragments of mAb 132 to rMoPrP by single-cycle kinetics. All sensorgrams were obtained using 2-fold serial dilutions of antibodies or rMoPrP: rFab-132, 0.63–10 and 1.25–20 nM; IgG-132, 0.31–5 and 0.625–10 nM; and rMoPrP, 3.13–50 and 6.25–100 nM. The dissociation constant was calculated from three independent experiments (mean ± SD). (C) Representative sensorgrams for the binding of authentic and recombinant antibody fragments of mAb 31C6 to rMoPrP by single-cycle kinetics. All sensorgrams were obtained using 2-fold serial dilutions of antibodies or rMoPrP: rFab-31C6, 0.06–1 and 0.13–2 nM; IgG-31C6, 3.13–50 and 6.25–100 pM; and rMoPrP, 0.16–2.5 and 0.31–5 nM.

The binding kinetics of mono- and bivalent mAbs 132 and 31C6 were analyzed using single-cycle kinetics analysis (Fig 6) and *k*_*a*_, *k*_*d*_, and *K*_*D*_ that were calculated from the sensorgrams are shown in Table 1. When rMoPrP was fixed on the sensorchip as a ligand and antibodies were used as analytes, under which IgG can bind bivalently to rMoPrP (Fig 6A, left), the *K*_*D*_ of rFab-132 was two orders of magnitude higher than that of IgG-132, indicating the lower reactivity of monovalent mAb 132 than bivalent form (Fig 6B, Table 1). However, a similar tendency was observed when the *K*_*D*_ of rFab-31C6 was compared with that of IgG-31C6 (Fig 6C, Table 1). These results suggest that the mechanism of PrP^Sc^-specific detection by mAb 132 is not simply explained by *K*_*D*_. As suggested by the multi-cycle kinetics in Fig 5, the binding rate of IgG-132 to rMoPrP, which is expressed as *k*_*a*_, was one order of magnitude lower than that of IgG-31C6 to rMoPrP (2.5 ± 1.1 × 10^5^ vs. 5.3 ± 1.4 × 10^6^ in Table 1); on the contrary, the dissociation rate of IgG-132 from rMoPrP, which is expressed as *k*_*d*_, was one order of magnitude lower than that of IgG-31C6 from rMoPrP when IgG was used as an analyte (1.2 ± 0.9 × 10^−5^ vs. 2.7 ± 0.8 × 10^−4^ in Table 1), suggesting that binding reaction of mAb 132 to rMoPrP proceed slowly compared to that of mAb 31C6. Although the *K*_*D*_ of the two rFab fragments or that of the two IgGs were comparable to each other, there are some differences in *k*_*a*_ and *k*_*d*_. The *k*_*a*_ of rFab-132 was 2.6 times higher than that of IgG-132 (6.4 × 10^5^ vs 2.5 × 10^5^ in Table 1). This difference was also observed in mAb 31C6: the *k*_*a*_ of rFab-31C6 was 1.6 times higher than that of IgG-31C6. By contrast, the *k*_*d*_ of rFab-132 (3.1 × 10^−3^) was approximately 260 times higher than that of IgG-132 (1.2 × 10^−5^), suggesting that the binding of rFab-132 is unstable compared with that of IgG-132. The difference in the *k*_*d*_ between rFab-31C6 and IgG-31C6 was smaller than that between rFab-132 and IgG-132; the *k*_*d*_ of rFab-31C6 (1.1 × 10^−2^) was only 40 times higher than that of IgG-31C6 (2.7 × 10^−4^). These results indicate that mAbs 132 and 31C6 intrinsically possess the similar binding affinity to rMoPrP. Although the monovalent binding of mAb 132 may be restricted by the kinetics limitation compared to that of mAb 31C6, the bivalent binding can significantly enhance the binding avidity of IgG-132.

**Table 1:** Summary of equilibrium and kinetic constants^a)^.

mAb	Ligand	Analyte	*K*_*D*_ (M)	*ka* (1/Ms)	*kd* (1/s)
mAb 132	rMoPrP	rFab-132	5.5 ± 2.9 × 10^−9^	6.4 ± 4.7 × 10^5^	3.1 ± 1.4 × 10^−3^
	rMoPrP	IgG-132	5.7 ± 2.3 × 10^−11^	2.5 ± 1.1 × 10^5^	1.2 ± 0.9 × 10^−5^
	IgG-132	rMoPrP	1.2 ± 0.2 × 10^−8^	6.3 ± 0.3 × 10^4^	7.6 ± 1.4 × 10^−4^
mAb 31C6	rMoPrP	rFab-31C6	1.3 ± 0.2 × 10^−9^	8.7 ± 0.3 × 10^6^	1.1 ± 0.2 × 10^−2^
	rMoPrP	IgG-31C6	5.2 ± 1.4 × 10^−11^	5.3 ± 1.4 × 10^6^	2.7 ± 0.8 × 10^−4^
	IgG-31C6	rMoPrP	2.0 ± 0.8 × 10^−10^	1.5 ± 0.2 × 10^7^	3.1 ± 1.7 × 10^−3^

^a)^ Calculated from sensorgrams measured by single-cycle kinetics. Mean ± SD from three independent experiments are shown.

The results described above suggest that antigen density influences the binding of mAb 132. To assess this possibility, rMoPrP was fixed on a CM5 sensorchip at 1.71 and 0.14 ng/mm^2^, and 10 nM IgG-132 and 1 nM IgG-31C6 were injected as analytes. The weight of bound antibodies was calculated from the RU of sensorgrams (Table 2). Due to the slow association rate of mAb 132, a concentration of mAb 132 10 times higher was used to obtain an RU level similar to that of mAb 31C6. When IgG-132 was used as an analyte, 30.8 pg/mm^2^ IgG-132 could bind to the sensorchip with 1.71 ng/mm^2^ rMoPrP. However, only 21.7% of IgG-132 (6.7 pg/mm^2^) could bind to the sensorchip with 0.14 ng/mm^2^ rMoPrP. In contrast to mAb 132, nearly 50% of IgG-31C6 still bound to sensorchip (22.7 ± 0.3 vs 11.3 ± 0.2 pg/mm^2^) even if the density of rMoPrP on the sensorchip decreased from 1.71 to 0.14 ng/mm^2^. These results indicate that the reaction of mAb 132 is more sensitive to antigen density than that of mAb 31C6.

**Table 2:** Antigen density-dependent binding of mAb 132.

Analyte (concentration^a)^)	Antibodies^b)^ (pg/mm^2^) bound to sensorchip with:	
	1.71 ng rMoPrP /mm^2^	0.14 ng rMoPrP /mm^2^
IgG-132 (10 nM)	30.8 ± 1.1^c)^	6.7 ± 0.1 ^c)^
IgG-31C6 (1 nM)	22.7 ± 0.3 ^c)^	11.3 ± 0.2 ^c)^

^a)^ Concentration of injected antibodies as analyte.

^b)^ Amounts of antibodies bound to sensorchip were estimated from sensorgrams as 1 RU = 1 pg/mm^2^.

^c)^ Mean ± SD from three independent experiments.

### Reactivity of mAb 132 to PrP^C^ crosslinked by anti-PrP mAb

**Fig 7:**
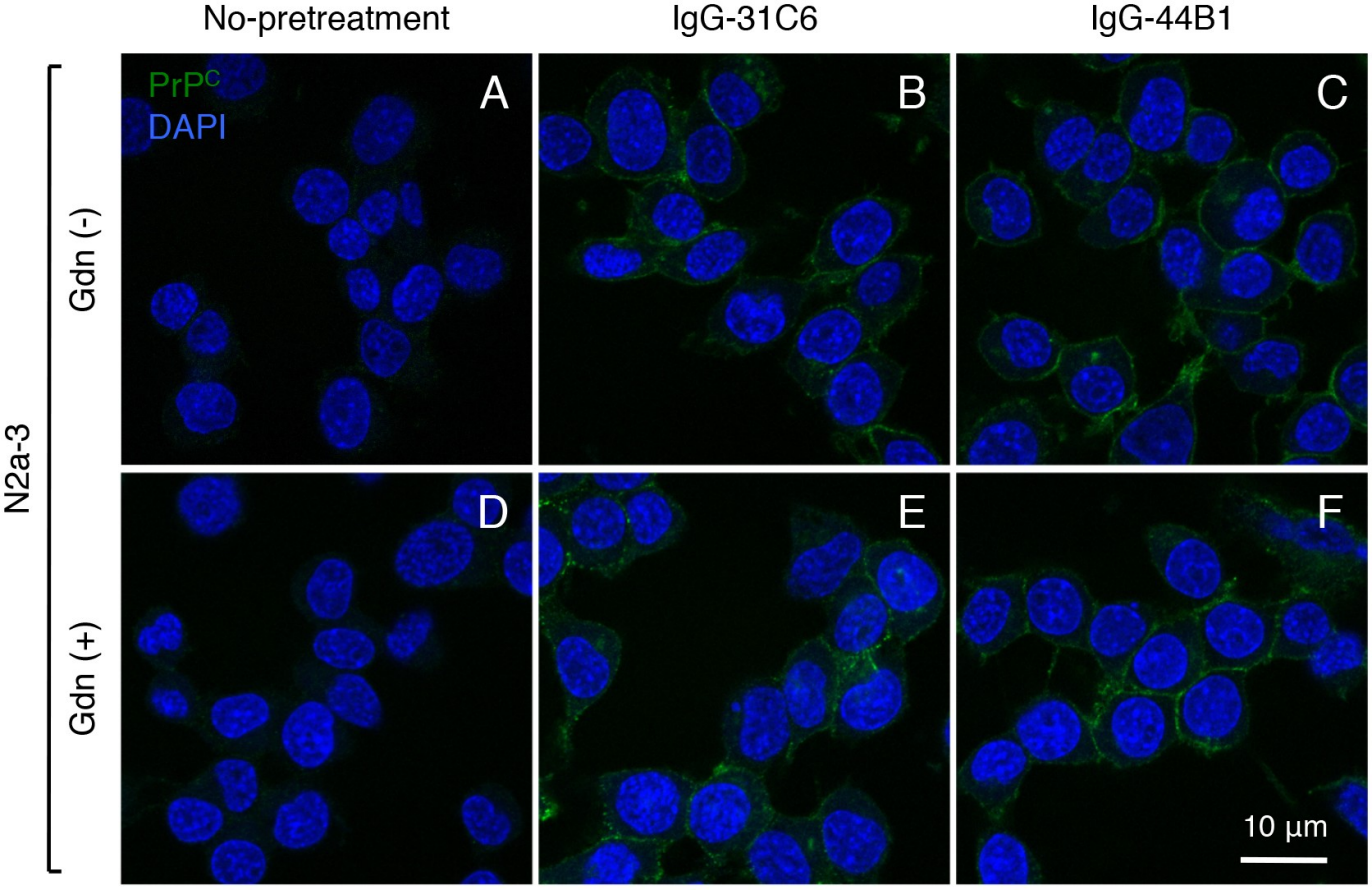
Detection of PrP^C^ on the surface of N2a-3 cells. N2a-3 cells were incubated with DMEM containing 10 nM IgG-31C6 (B, E) and IgG-44B1 (C, F) for 2 days. The cells were stained with 1 μg/ml Alexa Fluor 647-labeled mAb 132 after treatment with (D–F) or without (A–C) 5 M GdnSCN (green). The leftmost images show negative controls for antibody-treatment (A, D). Cell nuclei were stained with DAPI (blue).

The reactivity of mAb 132 and its derivatives suggest that a weak interaction in monovalent binding but the enhancement of binding avidity in bivalent binding accounts for apparent PrP^Sc^-specific detection in IFA. Therefore, we expected that mAb 132 would detect PrP^C^ on the cell surface if two PrP^C^ molecules existed close enough to allow antibody binding to PrP^C^ in its bivalent form. To address this possibility, PrP^C^ on the surface of N2a-3 cells was crosslinked by IgG-31C6 or IgG-44B1, which retains PrP^C^ molecules on the cell membrane possibly by crosslinking of two PrP^C^ molecules [26,28], and then stained with mAb 132 directly labeled with Alexa Flour 647. The mAb 132 detected PrP^C^ on the cell membrane of N2a-3 cells pretreated with IgG-31C6 or IgG-44B1 regardless of the GdnSCN treatment; however, mAb 132 did not react with PrP^C^ on the non-treated control N2a-3 cells (Fig 7 and S2 Fig). This result indicates that monovalent binding of mAb 132 to monomeric PrP^C^ is inefficient. However, PrP^Sc^ exists as oligomers, and thus the epitopes locate close enough to allow mAb 132 binding in a bivalent manner, which confers high binding avidity to mAb 132. This result also suggests that the epitope for mAb 132 is exposed on the surface of the PrP^C^ molecule.

### Detection of PrP^Sc^ in the brain section using mAb 132.

**Fig 8:**
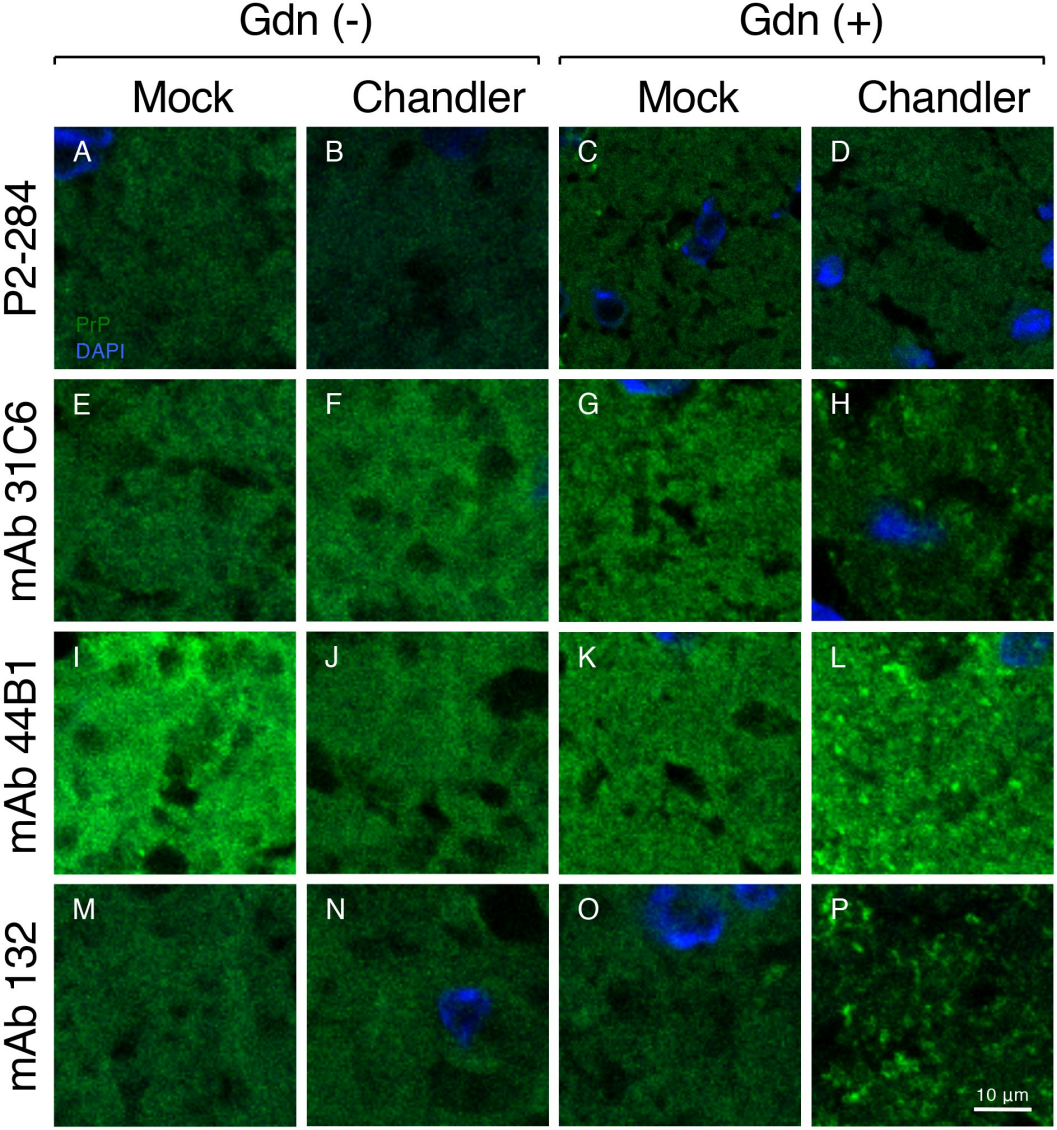
Detection of PrP^Sc^ in frozen brain sections. IFA was performed using the frozen brain sections of Chandler strain-infected (Chandler) and mock-infected (Mock) mice at 120 dpi with (Gdn(+)) or without (Gdn(-)) 2.5 M GdnSCN pretreatment. PrP molecules (green) were stained with mAbs 31C6, 44B1, and 132 followed by Alexa Fluor 488 F(ab’)_2_ fragment of goat anti-mouse IgG (H+L) (Invitrogen) as a secondary antibody. Anti-feline panleukopenia virus mAb P2-284 was used as a negative control mAb. Cell nuclei were counterstained with DAPI (blue). The fluorescent images were acquired from the coronal sections containing thalamus. Scale bar: 10 μm.

The reactivity of mAb 132 suggests that the inefficient monovalent binding decreases the signals from PrP^C^ to background level in IFA with cultured cells. To assess if the same mechanism could be applicable in tissue sections, particularly in brain where PrP^C^ abundantly expresses, we performed IFA using frozen brain sections from Chandler prion-infected and mock-infected mice (Fig 8). Compared to mAbs 31C6 (Fig 8E and 8G) and 44B1 (Fig 8I and 8K), mAb 132 showed weak fluorescence signals (Fig 8M and 8O) that are comparable to those by negative control mAb, P2-284 (Fig 8A and 8C), in the frozen section from mock-infected mouse, regardless of Gdn pretreatment. This indicates that mAbs 31C6 and 44B1 bind, whereas mAb 132 inefficiently binds to PrP^C^ expressed in the brain. After the Gdn pretreatment, these anti-PrP mAbs showed granular stains of PrP^Sc^ with intense fluorescence in the brain sections from the Chandler prion-infected mouse (Fig 8H, 8L and 8P). However, a fine analysis of PrP^Sc^ using mAbs 31C6 and 44B1 appears to be limited because of their reactivity to PrP^C^. In contrast, mAb 132 provides a better signal-noise-ratio for PrP^Sc^ detection because of the inefficient binding to PrP^C^. This is an advantage of mAb 132 for the analysis of PrP^Sc^ in tissue sections.

## Discussion

**Fig 9:**
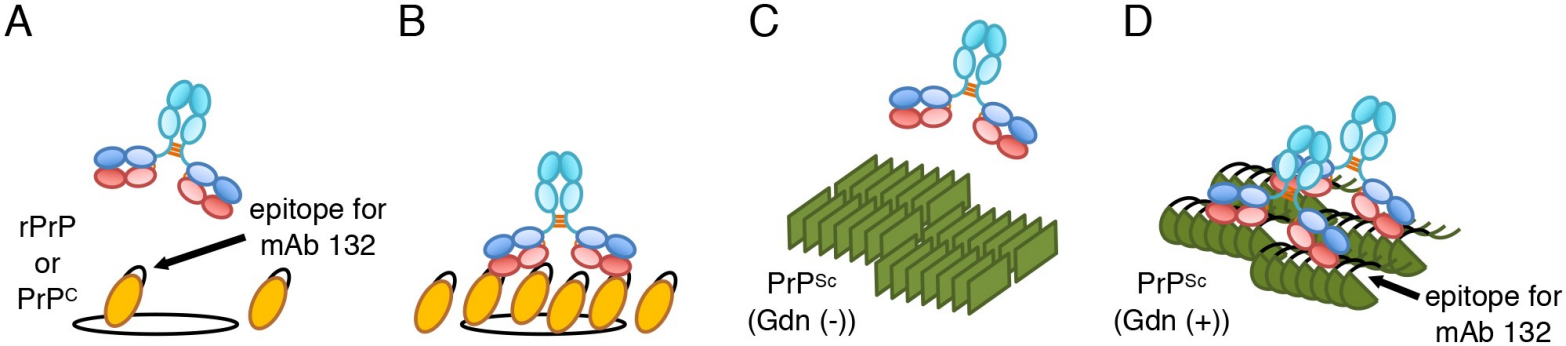
Mechanism for PrP^Sc^-specific detection by mAb 132. (A, B) Binding of mAb 132 to rPrP and PrP^C^ molecule. mAb 132 inefficiently binds to PrP molecule if density of PrP molecules is lower than that allows bivalent binding because of low affinity of monovalent binding of mAb 132 (A), whereas mAb 132 binds stably to PrP molecules by avidity effect if the density of PrP molecules is high enough to allow bivalent binding (B). Orange ovals: rPrP or PrP^C^; black lines on the orange ovals: epitope for mAb 132. Circles indicates the area where bivalent binding of intact mouse IgG_1_ takes place. (C, D) Binding of mAb 132 to PrP^Sc^ oligomer/aggregate. mAb 132 does not bind to PrP^Sc^ without guanidinium salt pretreatment (C, Gdn (-)), whereas epitopes for mAb 132 are exposed by the guanidinium salt pretreatment (D, Gdn (+)), multiple epitopes for mAb 132 on the PrP^Sc^ oligomer/aggregate allow bivalent binding of mAb 132 (Gdn (+)).

Although mAb 132 is a pan-PrP antibody, the use of this mAb facilitates reliable PrP^Sc^-specific staining in cells or tissue sections pretreated with a chaotropic reagent [18–20,32]. However, the mechanism of PrP^Sc^-specific detection by mAb 132 is largely unclear. To investigate this mechanism, we analyzed the reactivity and binding kinetics of mono- and bivalent mAb 132 using ELISA and SPR. The monovalent binding of rFab-132 was significantly weaker than that of IgG-132, whereas that of rFab of mAbs 31C6 and 44B1 was as efficient as authentic IgG (Fig 2A and 2B). These results indicate that bivalent binding requires efficient binding of mAb 132. Consistent with the results of ELISA (Figs 2 and 3D), analyses of binding kinetics by SPR showed that antigen density influences mAb 132 binding (Table 2). Crystallographic analysis of IgG revealed that the distance between two Fab domains of intact mouse IgG_1_ is around 11.8 nm [33]. The number of rMoPrP molecules in a circle of 11.8 nm in diameter can be estimated as 6.6 and 3.7 molecules in case of 100 ng/well rMoPrP in ELISA (Fig 2) and 1.71 ng/mm^2^ rMoPrP in SPR (Table 2), respectively. Thus, theoretically, the rMoPrP density in these conditions appears high enough to allow bivalent binding of mAb 132 [34]. On the other hand, for 12.5 ng/well rMoPrP, the lowest concentration that mAb 132 could bind in ELISA (Fig 2B), and 0.14 ng/mm^2^ rMoPrP in SPR, the number of rMoPrP molecules can be estimated as 0.9 and 0.3, respectively, in a circle of 11.8 nm in diameter. Thus, it is expected that monovalent binding of IgG to rMoPrP occurred mainly under these conditions (Fig 9A). The estimated numbers of rMoPrP molecules are consistent with the idea that bivalent binding confers a higher binding stability to mAb 132 (Fig 9B). Indeed, compared with IgG-132, binding of rFab-132 to rMoPrP in ELISA was not so efficient even at the highest antigen concentration (100 ng/well rMoPrP) (Figs 2 and 3D). This trend was not observed with other pan-PrP mAbs, 31C6 and 44B1, and their rFab fragments. Furthermore, the amount of IgG-132 that bound to the sensorchip with 0.14 ng/mm^2^ rMoPrP was only 20% of that bound to the sensorchip with 1.71 ng/mm^2^ rMoPrP. Taken together, these results indicate that monovalent mAb 132 binding is less stable than bivalent mAb 132 binding.

In the present study, monovalent and bivalent forms of mAbs 132 and 31C6 showed similar *K*_*D*_ values when they were used as analytes. However, mAb 132 possessed the smaller *k*_*a*_ and *k*_*d*_ values in one order of magnitude than those of mAb 31C6 regardless of the valency (Table 1). A small *k*_*a*_ value indicates slow binding rate of mAb to its antigens. It has been reported that mAb with a smaller *k*_*a*_ required longer reaction time and relatively high concentration, compared to that with a larger *k*_*a*_ [35]. Thus, the inefficient binding of rFab-132 (Figs 2, 3D and 4) may be explained by the kinetic limitation at the lower concentration and/or in the short time incubation. However, rFab-132 showed only 2.6 times higher *k*_*a*_ values than IgG-132 (Table 1), suggesting that the PrP^Sc^-specific staining of mAb 132 is not explained by association kinetics in different valency. On the other hand, a small *k*_*d*_ value indicates slow dissociation rate of mAb that binds to antigens. The *k*_*d*_ of rFab-132 was approximately 260 times larger than that of IgG-132, whereas rFab-31C6 showed 40 times larger *k*_*d*_ than IgG-31C6 (Table 1). The difference in the *k*_*d*_ between mono- and bivalent mAb 132 was 6.5 times higher than that of mAb 31C6. Avidity is applied to the sum of the binding affinity of antibody to epitopes crosslinked by bivalent binding, which is an ability inherent to the epitope and depends on its density [34,36]. Thus, it is expected that the bivalent binding of IgG-132 may strongly enhance the retention on the surface of antigens compared to that of IgG-31C6. Indeed, the enhancement of the antitumor and antiviral activity through the bivalent binding have been shown in anti-EGFR nimotuzumab [37], and anti-influenza virus mAb S139/1 [38] and anti-Dengue virus mAb E106 [39]. Taken together, the antigen density-dependent enhancement of the binding by avidity effect may be a key mechanism of PrP^Sc^-specific detection by mAb 132.

MAb 132 bound to PrP^C^ expressed in N2a-3 cells when PrP^C^ molecules were crosslinked by anti-PrP antibodies (Fig 7). This result clearly indicates that bivalent binding confers a high binding avidity to mAb 132. Additionally, detection does not require the pretreatment of cells with guanidinium salt, indicating that the epitope for mAb 132 locates an accessible surface of the PrP^C^ (Fig 9A and 9B). In contrast, guanidinium salt pretreatment is essential for PrP^Sc^ detection. This means that the epitope for mAb 132 in the PrP^Sc^ molecule is inaccessible to antibodies before denaturation (Fig 9C). However, PrP^Sc^ exists as an oligomer of the PrP molecule so that more than two epitopes for mAb 132, which allow antibodies to bind to PrP^Sc^ oligomers in a bivalent manner, became accessible after denaturation (Fig 9D). Thus, weak monovalent binding of mAb 132 to monomeric PrP^C^ diminishes the signals from PrP^C^ to background level, whereas after exposure of the epitope, mAb 132 selectively binds to oligomeric PrP^Sc^ in a bivalent manner (Figs 8, 9A and 9D,). This combination provides a better signal-noise-ratio and therefore enables reliable PrP^Sc^ detection in cells and sections using IFA, cell-based ELISA, and flow cytometry (Fig 8) [18,20,21,32]. However, considering the mechanism of PrP^Sc^-specific detection of mAb 132 described above, mAb 132 will react with PrP^C^ if more than two PrP^C^ molecules exist within the distance that allows bivalent binding of IgG (Fig 9B).

MAb 132 cannot bind with the epitope on PrP^Sc^ molecules without the guanidinium salt pretreatment [20]. A group of anti-PrP antibodies that recognize the region around aa 90–120 of PrP molecules exhibit a similar property [40–42]. Thus, the region is partially or completely buried in PrP^Sc^ molecules. Moreover, H/D exchange mass spectrometry showed that the solvent exposure of the hydrophobic region comprised aa 111–120, including the epitope for mAb 132 on PrP^Sc^, was significantly decreased compared with that of rMoPrP or PrP^Sc^ intermediates [43]. These results are consistent with the fact that mAb 132 cannot directly access the epitope on PrP^Sc^ molecules. Most PrP^Sc^-specific mAbs recognize the conformational epitopes that are exposed on PrP^Sc^ molecules, and thus, those antibodies react with PrP^Sc^ without denaturation. In contrast to the reactivity of mAb 132 to PrP^Sc^, the reactivity of PrP^Sc^-specific mAbs decreased after denaturation of PrP^Sc^ molecules [8,10,13,44].

In the current study, we elucidated the mechanism of PrP^Sc^-specific detection by mAb 132. This mAb belongs to a group of pan-PrP antibodies that cannot distinguish PrP^Sc^ from PrP^C^; however, the antigen density-dependent enhancement of binding stability via bivalent binding facilitates the detection of PrP^Sc^ because PrP^Sc^ exists as oligomer and/or aggregates of PrP molecules. PrP^Sc^ oligomers consisting of small numbers of PrP molecules are sensitive to PK treatment [7], so the finding in this study provides a mechanistic rationale for the detection of PrP^Sc^-sen in prion-infected cells by mAb 132 [22]; guanidinium salt treatment exposed epitopes for mAb 132 on PrP^Sc^ molecules, which are close enough to allow bivalent binding of mAb 132. The epitope for mAb 132 is widely conserved across species from mammals to chickens [45,46]; therefore, mAb 132 will be useful for the detection of PrP^Sc^ in various species including humans.

## Supporting information

S1 Fig Strategy for molecular cloning of antibodies and construction of the expression plasmids. Detail description is shown in S1 Text.
(PPTX)

S2 Fig Detection of PrP^C^ and anti-PrP mAbs used for crosslinking in N2a-3 cells. Detail description is shown in S1 Text.
(PPTX)

S1 Table Specific primers for the cloning of antibodies. (DOC)

S2 Table Specific primers for the cloning of monovalent antibodies. (DOC)

S3 Table Specific primers for construction of fusion proteins. (DOC)

S1 Text Documents for supporting information. (DOC)
