# Supplementary material for: Enhancement of binding avidity by bivalent binding enables PrPSc-specific detection by anti-PrP monoclonal antibody 132
Source: PLoS One. 2019 Jun 6;14(6):e0217944. doi: 10.1371/journal.pone.0217944 (PMC6553756; doi:10.1371/journal.pone.0217944)
Supplement: S1 Fig — Detail description is shown in S1 Text. (PPTX) [file pone.0217944.s001.pptx]

## Slide 1
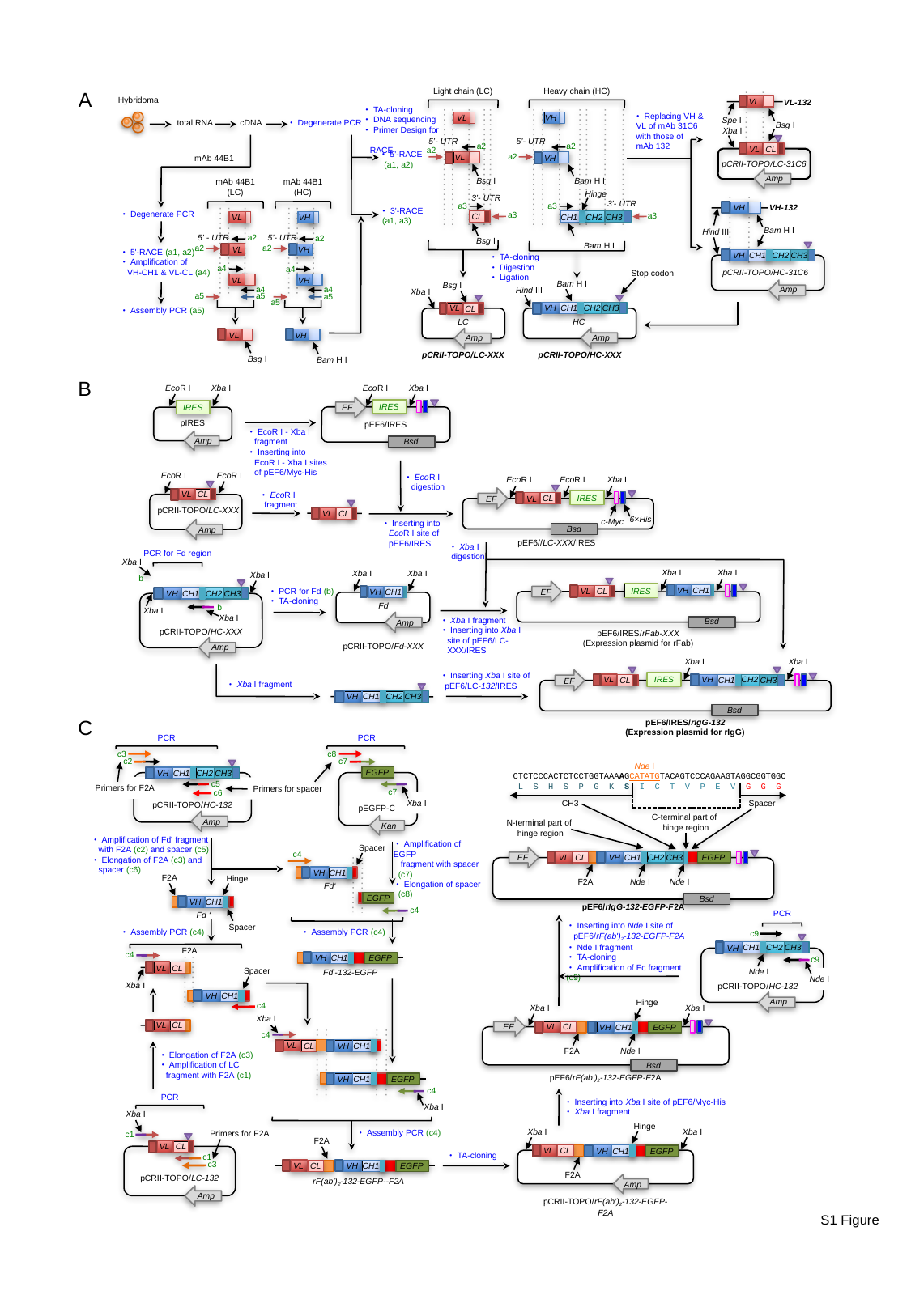

Light chain (LC)
Heavy chain (HC)
A
Hybridoma
VL
VL-132
・TA-cloning
・DNA sequencing
・Primer Design for
 RACE
Spe I
・Replacing VH &
 VL of mAb 31C6
 with those of
 mAb 132
VL
VH
Bsg I
・Degenerate PCR
total RNA
cDNA
Xba I
5’- UTR
5’- UTR
a2
a2
VL
CL
Amp
pCRII-TOPO/LC-31C6
a2
・5’-RACE
 (a1, a2)
a2
VL
Bsg I
mAb 44B1
VH
Bam H I
mAb 44B1
(LC)
mAb 44B1
(HC)
Hinge
3’- UTR
3’- UTR
a3
a3
VH-132
VH
・3’-RACE
 (a1, a3)
・Degenerate PCR
a3
a3
CL
CH1
CH2
CH3
VH
VL
Hind III
Bam H I
Bam H I
Bsg I
5' - UTR
5’- UTR
a2
a2
a2
a2
VL
VH
・5'-RACE (a1, a2)
・Amplification of
 VH-CH1 & VL-CL (a4)
VH
CH1
CH2
CH3
・TA-cloning
・Digestion
・Ligation
a4
a4
pCRII-TOPO/HC-31C6
Stop codon
Bam H I
Hind III
VH
CH1
CH2
CH3
HC
Amp
pCRII-TOPO/HC-XXX
CL
VH
VL
Bsg I
Xba I
VL
CL
LC
Amp
pCRII-TOPO/LC-XXX
a4
a4
Amp
a5
a5
a5
a5
・Assembly PCR (a5)
VL
Bsg I
VH
Bam H I
B
EcoR I
Xba I
IRES
EF
pEF6/IRES
Bsd
EcoR I
Xba I
IRES
pIRES
Amp
・EcoR I - Xba I
 fragment
・Inserting into
 EcoR I - Xba I sites
 of pEF6/Myc-His
EcoR I
EcoR I
VL
CL
Amp
pCRII-TOPO/LC-XXX
・EcoR I
 digestion
EcoR I
EcoR I
Xba I
CL
VL
IRES
EF
6×His
c-Myc
Bsd
pEF6//LC-XXX/IRES
・EcoR I
 fragment
VL
CL
・Inserting into
 EcoR I site of
 pEF6/IRES
・Xba I
 digestion
PCR for Fd region
Xba I
Xba I
Xba I
VL
CL
VH
CH1
IRES
EF
Bsd
pEF6/IRES/rFab-XXX
(Expression plasmid for rFab)
Xba I
Xba I
CH1
VH
Fd
Amp
pCRII-TOPO/Fd-XXX
Xba I
b
・PCR for Fd (b)
・TA-cloning
VH
CH1
CH2
CH3
Xba I
b
Xba I
・Xba I fragment
・Inserting into Xba I
 site of pEF6/LC-
 XXX/IRES
pCRII-TOPO/HC-XXX
Amp
Xba I
Xba I
VL
CL
IRES
EF
Bsd
VH
CH2
CH3
CH1
pEF6/IRES/rIgG-132
(Expression plasmid for rIgG)
・Inserting Xba I site of
 pEF6/LC-132/IRES
・Xba I fragment
VH
CH1
CH2
CH3
C
PCR
PCR
c3
c8
c7
c2
 Nde I
CTCTCCCACTCTCCTGGTAAAAGCATATGTACAGTCCCAGAAGTAGGCGGTGGC
 L S H S P G K S I C T V P E V G G G
VH
CH1
CH2
CH3
EGFP
Primers for F2A
c5
Primers for spacer
c7
c6
CH3
Spacer
C-terminal part of
hinge region
Xba I
pCRII-TOPO/HC-132
pEGFP-C1
Amp
N-terminal part of
hinge region
Kan
・Amplification of Fd' fragment
 with F2A (c2) and spacer (c5)
・Elongation of F2A (c3) and
 spacer (c6)
・Amplification of EGFP
 fragment with spacer
 (c7)
・Elongation of spacer
 (c8)
Spacer
c4
VL
CL
CH2
CH3
CH1
VH
EGFP
EF
F2A
Nde I
Nde I
Bsd
pEF6/rIgG-132-EGFP-F2A
VH
CH1
F2A
Hinge
Fd‘
EGFP
VH
CH1
c4
PCR
Fd ‘
Spacer
・Inserting into Nde I site of
 pEF6/rF(ab')2-132-EGFP-F2A
・Nde I fragment
・TA-cloning
・Amplification of Fc fragment (c9)
・Assembly PCR (c4)
・Assembly PCR (c4)
c9
CH1
CH2
CH3
VH
F2A
c4
VH
CH1
EGFP
c9
Nde I
VL
CL
Spacer
Fd’-132-EGFP
Nde I
Xba I
pCRII-TOPO/HC-132
VH
CH1
Amp
Hinge
Xba I
Xba I
EF
VL
CL
VH
CH1
EGFP
F2A
Nde I
Bsd
pEF6/rF(ab’)2-132-EGFP-F2A
c4
Xba I
VL
CL
c4
VL
CL
VH
CH1
・Elongation of F2A (c3)
・Amplification of LC
 fragment with F2A (c1)
VH
CH1
EGFP
c4
PCR
・Inserting into Xba I site of pEF6/Myc-His
・Xba I fragment
Xba I
Xba I
Hinge
Xba I
Xba I
VL
CL
VH
CH1
EGFP
F2A
Amp
pCRII-TOPO/rF(ab’)2-132-EGFP-F2A
・Assembly PCR (c4)
Primers for F2A
c1
F2A
VL
CL
・TA-cloning
c1
pCRII-TOPO/LC-132
c3
VL
CL
VH
CH1
EGFP
rF(ab’)2-132-EGFP--F2A
Amp
S1 Figure

## Slide 2
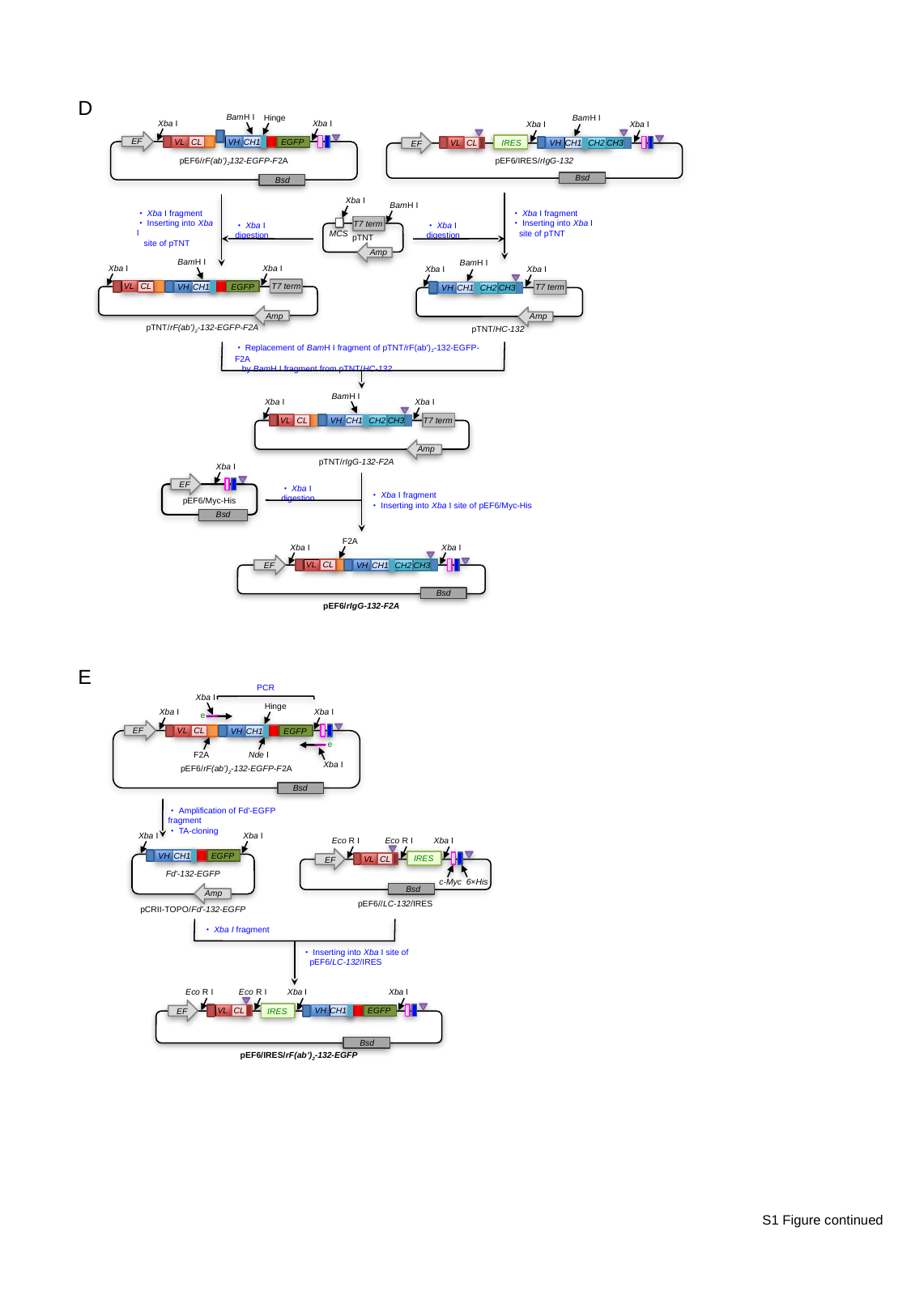

D
BamH I
Hinge
Xba I
Xba I
EF
VL
CL
VH
CH1
EGFP
pEF6/rF(ab’)2132-EGFP-F2A
Bsd
BamH I
Xba I
Xba I
VL
CL
IRES
EF
Bsd
VH
CH1
CH2
CH3
pEF6/IRES/rIgG-132
Xba I
BamH I
T7 term
MCS
pTNT
Amp
・Xba I fragment
・Inserting into Xba I
 site of pTNT
・Xba I fragment
・Inserting into Xba I
 site of pTNT
・Xba I digestion
・Xba I digestion
BamH I
Xba I
Xba I
T7 term
VL
CL
VH
CH1
EGFP
Amp
pTNT/rF(ab’)2-132-EGFP-F2A
BamH I
Xba I
Xba I
VH
CH1
CH2
CH3
T7 term
Amp
pTNT/HC-132
・Replacement of BamH I fragment of pTNT/rF(ab')2-132-EGFP-F2A
 by BamH I fragment from pTNT/HC-132
BamH I
Xba I
Xba I
VH
CH1
CH2
CH3
VL
CL
T7 term
Amp
pTNT/rIgG-132-F2A
Xba I
EF
pEF6/Myc-His
Bsd
・Xba I digestion
・Xba I fragment
・Inserting into Xba I site of pEF6/Myc-His
F2A
Xba I
Xba I
VH
CH1
CH2
CH3
VL
CL
EF
Bsd
pEF6/rIgG-132-F2A
E
PCR
Xba I
Hinge
Xba I
Xba I
EF
VL
CL
VH
CH1
EGFP
F2A
Nde I
pEF6/rF(ab’)2-132-EGFP-F2A
e
e
Xba I
Bsd
・Amplification of Fd'-EGFP fragment
・TA-cloning
Xba I
Xba I
VH
CH1
EGFP
Fd'-132-EGFP
Amp
pCRII-TOPO/Fd'-132-EGFP
Eco R I
Eco R I
Xba I
VL
CL
IRES
EF
c-Myc
6×His
Bsd
pEF6//LC-132/IRES
・Xba I fragment
・Inserting into Xba I site of
 pEF6/LC-132/IRES
Eco R I
Eco R I
Xba I
Xba I
VL
CL
VH
CH1
EGFP
EF
IRES
Bsd
pEF6/IRES/rF(ab’)2-132-EGFP
S1 Figure continued
