# Supplementary material for: Enhancement of binding avidity by bivalent binding enables PrPSc-specific detection by anti-PrP monoclonal antibody 132
Source: PLoS One. 2019 Jun 6;14(6):e0217944. doi: 10.1371/journal.pone.0217944 (PMC6553756; doi:10.1371/journal.pone.0217944)
Supplement: S2 Fig — Detail description is shown in S1 Text. (PPTX) [file pone.0217944.s002.pptx]

## Slide 1
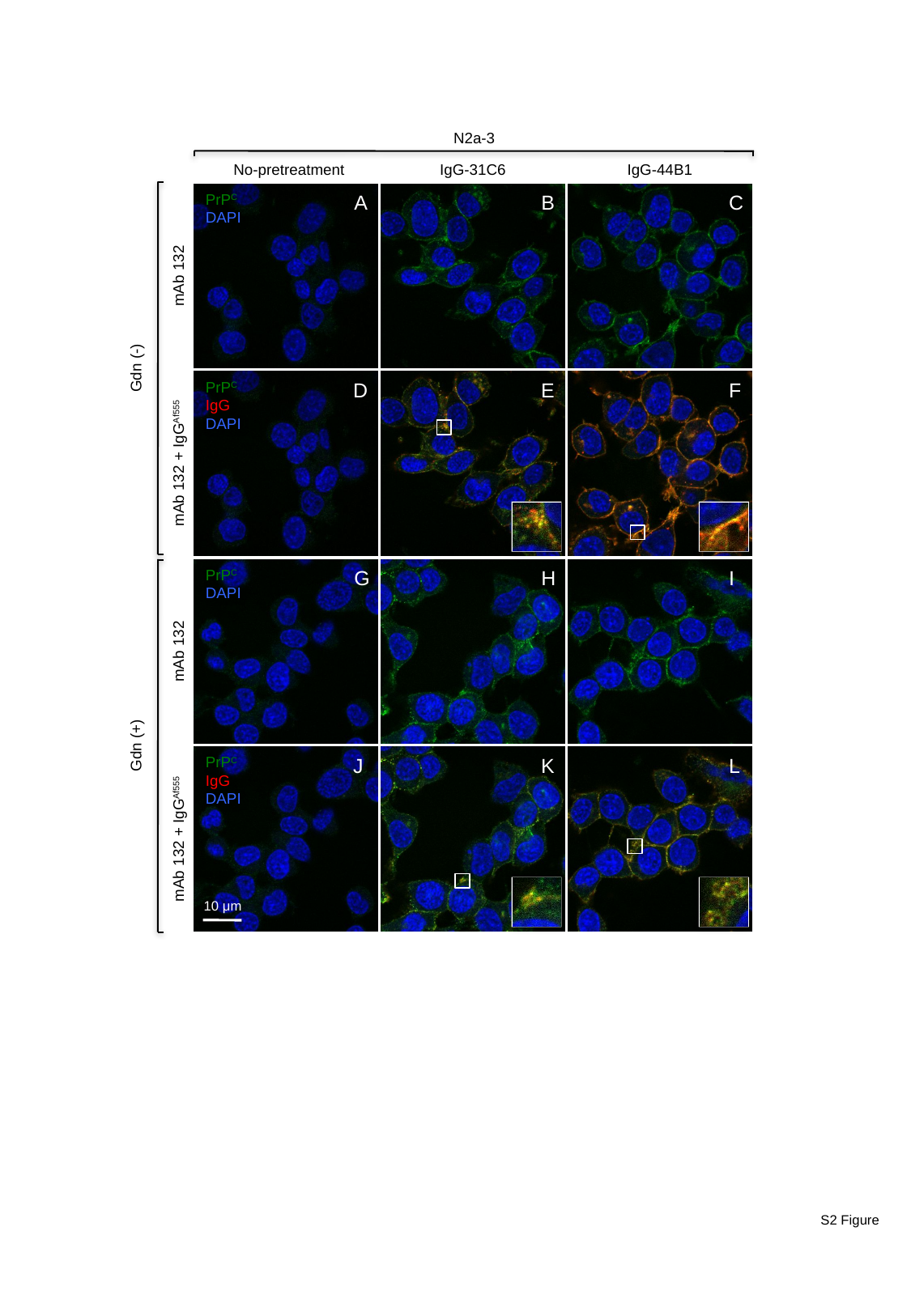

N2a-3
No-pretreatment
IgG-31C6
IgG-44B1
 PrPC
 DAPI
A
B
C
mAb 132
Gdn (-)
 PrPC
 IgG
 DAPI
D
E
F
mAb 132 + IgGAf555
 PrPC
 DAPI
G
H
I
mAb 132
Gdn (+)
 PrPC
 IgG
 DAPI
J
K
L
mAb 132 + IgGAf555
10 μm
S2 Figure
