# Supplementary material for: Enhancement of binding avidity by bivalent binding enables PrPSc-specific detection by anti-PrP monoclonal antibody 132
Source: PLoS One. 2019 Jun 6;14(6):e0217944. doi: 10.1371/journal.pone.0217944 (PMC6553756; doi:10.1371/journal.pone.0217944)
Supplement: S1 Table — (DOC) [file pone.0217944.s003.doc]

**S1 Table.** **Specific primers for the cloning of antibodies.**

| Constructs | Primer names^*^ | Sequences |
| --- | --- | --- |
| mAb 31C6 | 31C6VHF1^a3^ | 5’-CTGTCTATCCACTGGCCCCT-3’ |
|  | 31C6VHF2^a3^ | 5’-ACCCTGGGATGCCTGGTCAA-3’ |
|  | 31C6VHR1^a1, a2^ | 5’-CGTGGTGTGCTGCTGGCCGGGT-3’ |
|  | 31C6VHR2^a2^ | 5’-CTGGCTGGGCCAGGTGCTGG-3’ |
|  | 31C6VHR3^a2^ | 5’-CTGGGAAGGTGTGCACACCG-3’ |
|  | 31C6VLF1^a3^ | 5’-GGAGTCCCATCAAGGTTCAG-3’ |
|  | 31C6VLR1^a1, a2^ | 5’-CGTGGTGCTGCTGGCCGGGT-3’ |
|  | 31C6VLR2^a2^ | 5’-GCCAGTGGATAGACTGATGG-3’ |
|  | 31C6VLR3^a2^ | 5’-TTGACCAGGCATCCCAGAGT-3’ |
| mAb 44B1 | ANC3’_SLIC^a2, a5^ | 5’-AATCTAGACTAAAGAATTCCAGTCAGTCAGTCATAGTC-3’ |
|  | 44B1VHF1^a3^ | 5’-CCCTGTGTGTGGAGGTACAA-3’ |
|  | 44B1VHR1^a1^ | 5’-CGCGGTGTTGCTGGCCGGG-3’ |
|  | 44B15’VHR1^a1^ | 5’-GGAGTTACTTGTACAGTA-3’ |
|  | 44B15’VHR2^a2^ | 5’-GGTGTTCCTGGCATTGTCTC-3’ |
|  | 44B15’VHR3^a2^ | 5’-TGGTGAATCGGCCCTTCACA-3’ |
|  | 44B1VHR2^a4^ | 5’-GGCTGGGCCAGGTGTTCGAG-3’ |
|  | 44B1VHR3^a4, a5^ | 5’-GACTGCAGGAGAGCTGGGAA-3’ |
|  | 44B1VLF1^a3^ | 5’-AGTGGGTCTAGGACAGACTT-3’ |
|  | 44B15’VLR1^a1^ | 5’-CCCCTCCGAATGTATACGGA-3’ |
|  | 44B15’VLR2^a2^ | 5’-GGTGAAGTCTGTCCTACACC-3’ |
|  | 44B15’VLR3^a2^ | 5’-GCCACTGAACCTGGCAGGGA-3’ |
|  | 31C6VLR2^a4^ | 5’-GCCAGTGGATAGACTGATGG-3’ |
|  | 31C6VLR3^a4, a5^ | 5’-TTGACCAGGCATCCCAGAGT-3’ |
| mAb 132^#^ | ANC3’_SLIC^a2^ | 5’-AATCTAGACTAAAGAATTCCAGTCAGTCAGTCATAGTC-3’ |
|  | 31C6VHF1^a3^ | 5’-CTGTCTATCCACTGGCCCCT-3’ |
|  | 31C6VHF2^a3^ | 5’-ACCCTGGGATGCCTGGTCAA-3’ |
|  | 31C6VHR1^a1, a2^ | 5’-CGTGGTGTGCTGCTGGCCGGGT-3’ |
|  | 31C6VHR2^a2^ | 5’-CTGGCTGGGCCAGGTGCTGG-3’ |
|  | 31C6VLR1^a1, a2^ | 5’-CGTGGTGCTGCTGGCCGGGT-3’ |
|  | 31C6VLR3^a2^ | 5’-TTGACCAGGCATCCCAGAGT-3’ |
|  | 31C6VLF1^a3^ | 5’-GGAGTCCCATCAAGGTTCAG-3’ |
|  | 132VLF^a3^ | 5’-GCCTGAAGATTTTGGGAGTT-3’ |

*: Superscript letters in primer name are corresponding to the steps of 5’ or 3’ RACE, or assembly PCR shown in S1 Fig A. The detailed description is as follow:

a1: Primers used for the cDNA first strand synthesis for 5’ RACE. The primer for 3’-RACE (dTAnc) has been shown elsewhere .

a2: Primers for 5’ RACE. The gene fragments encoding the 5’-terminal region of each chain were amplified by nested PCR. The forward primers for mAb 31C6 (ANC1, 3) and mAbs 44B1, 132 (ANC1) have been shown elsewhere . The ANC3’_SLIC is modified primer of ANC3 including *Xba* I site (under lined sequence).

a3: Primers for 3’ RACE. The 3’-terminal gene fragment of each heavy and light chain was amplified by nested PCR. The reverse primers (dTAnc1, 2) have been shown elsewhere .

a4: Gene fragments encoding from variable region to constant region (VH-CH1, VL-CL) of mAb 44B1 were separately amplified using these primers with forward primers, V_Κc_ or V_Hd_ .

a5: Assembly-PCR primers for the 5’-termnal gene fragments of mAb 44B1. After assembling the product of 5’ RACE and amplification of variable-constant region, assembled gene fragments only including the 5’-UTR of mAb 44B1 were amplified.

#: Since mAbs 31C6 and 132 share the nucleotide sequence of constant regions, same primers were used for the amplification.
