# Supplementary material for: Enhancement of binding avidity by bivalent binding enables PrPSc-specific detection by anti-PrP monoclonal antibody 132
Source: PLoS One. 2019 Jun 6;14(6):e0217944. doi: 10.1371/journal.pone.0217944 (PMC6553756; doi:10.1371/journal.pone.0217944)
Supplement: S2 Table — (DOC) [file pone.0217944.s004.doc]

**S2 Table.** **Specific primers for the cloning of monovalent antibodies.**

| Constructs | Primer names^*^ | Sequences^#^ |
| --- | --- | --- |
| rFab-31C6 | ANC3’_SLIC^b^ | 5’-AATCTAGACTAAAGAATTCCAGTCAGTCAGTCATAGTC-3’ |
|  | 31C6Fd_R^b^ | 5’-TTTCTAGAACCACAATCCCTGGGCACAATTTTCTT-3’ |
| rFab-44B1 | ANC3’_SLIC^b^ | 5’-AATCTAGACTAAAGAATTCCAGTCAGTCAGTCATAGTC-3’ |
|  | 44B1Fd_R^b^ | 5’-AATCTAGACTGTGTTATGGGCACTC-3’ |
| rFab-132 | ANC3’_SLIC^b^ | 5’-AATCTAGACTAAAGAATTCCAGTCAGTCAGTCATAGTC-3’ |
|  | 132Fd_R^b^ | 5’-TTTCTAGAACCACAATCCCTGGGCACAATTTTCTT-3’ |
| rFab-132-F2A^$^ | 132LC_F^c1, c4^ | 5’-AATCTAGAATGAGTGTGCTCACTCAGGTCCTG-3’ |
|  | 132LC_2A_R^c1^ | 5’-CAACTTGAGAAGGTCAAAATTCAAAGTCTGTTTCACACACTCATTCCTGTT-3’ |
|  | F2A_R1^c3^ | 5’-CAACTTGAGAAGGTCAAAATTCAAAGTCTGTTT-3’ |
|  | F2A_R2^c3^ | 5’-CTCCACGTCCTCCCGCCAACTTGAGAAGGTCAAA-3’ |
|  | F2A_F^c3^ | 5’-TGGCGGGAGACGTGCACTCCAACCCAGGGCCCA-3’ |
|  | 132Fd_2A_F^c2^ | 5’-TCCAACCAGGGCCCATGGGATGGAGCTGTATC-3’ |
|  | 132Fd_Myc_R^c2, c4^ | 5’-ACCAGAACCGCCACCGCCTGATACTTCTGGGAC-3’ |

*: Superscript letters in primer name are corresponding to the steps of PCR shown in S1 Fig B and C. The detailed description is as follow:

b: Primers for the amplification of Fd region of mAbs 31C6, 44B1 and 132 (S1 Fig B).

c1 and c2: Primers used for the amplification of gene fragment including LC with F2A portion (c1), Fd region with F2A portion (c2) (S1 Fig C).

c3: Primers used for the elongation of F2A portion of the fragment of LC and Fd with F2A (S1 Fig C).

c4: Primers for the assembly PCR to amplify the fusion genes with F2A (S1 Fig C).

#: Under line in the sequences indicate *Xba* I site.

$: We could not obtain any expression plasmid for rFab-F2A, because the constructs never cloned without irregular single base addition in the F2A sequence even though the same primers for construction of rF(ab’)_2_-132-EGFP (S3 Table) were used.
