# Supplementary material for: Enhancement of binding avidity by bivalent binding enables PrPSc-specific detection by anti-PrP monoclonal antibody 132
Source: PLoS One. 2019 Jun 6;14(6):e0217944. doi: 10.1371/journal.pone.0217944 (PMC6553756; doi:10.1371/journal.pone.0217944)
Supplement: S3 Table — (DOC) [file pone.0217944.s005.doc]

**S3 Table.** **Specific primers for construction of fusion proteins.**

| Constructs | Primer names^*^ | Sequences^$^ |
| --- | --- | --- |
| rFab-132-EGFP | ANC3’_SLIC^c0^ | 5’-AATCTAGACTAAAGAATTCCAGTCAGTCAGTCATAGTC-3’ |
|  | 132EGFP1_R^c0^ | 5’-CTTGCTCACACCACAATCCCTGGGCACAAT-3’ |
|  | 132EGFP1_F^c7^ | 5’-CCCAGGGATTGTGGTGTGAGCAAGGGCGAG-3’ |
|  | EGFP_R^c7^ | 5’-AATCTAGACTTGTACAGCTCGTCCATGCCGAGAGT-3’ |
| rF(ab’)_2_-132-  EGFP | 132LC_F^c1, c4^ | 5’-AATCTAGAATGAGTGTGCTCACTCAGGTCCTG-3’ |
|  | 132LC_2A_R^c1^ | 5’-CAACTTGAGAAGGTCAAAATTCAAAGTCTGTTTCACACACTCATTCCTGTT-3’ |
|  | F2A_R1^c3^ | 5’-CAACTTGAGAAGGTCAAAATTCAAAGTCTGTTT-3’ |
|  | F2A_R2^c3^ | 5’-CTCCACGTCCTCCCGCCAACTTGAGAAGGTCAAA-3’ |
|  | F2A_F^c3, c4^ | 5’-TGGCGGGAGACGTGCACTCCAACCCAGGGCCCA-3’ |
|  | 132Fd_2A_F^c2^ | 5’-TCCAACCAGGGCCCATGGGATGGAGCTGTATC-3’ |
|  | 132Fd_G4S_R^c5^ | 5’-ACCAGAACCGCCACCGCCTGATACTTCTGGGAC-3’ |
|  | Gly4Ser_R^c4, c6^ | 5’-ACCGCCGGAGCCACCGCCACCAGAACCGCCACC-3’ |
|  | G4S_EGFP_F1^c7^ | 5’-GGCGGTTCTGTGAGCAAGGGCGAGGAGCTGTTC-3’ |
|  | G4S_EGFP_F2^c8^ | 5’-GGTGGCTCCGGCGGTGGCGGTTCTGTGAGCAAG-3’ |
|  | EGFP_R^c4, c7, e^ | 5’-AATCTAGACTTGTACAGCTCGTCCATGCCGAGAGT-3’ |
|  | 132Fd_EGFP_F^e^ | 5’-AATCTAGAATGGGATGGAGCTGTATCATCCTCTTT-3’ |
| rIgG(ΔCH3)-  132-EGFP | 132Fc_F^c9^ | 5’-AAGCCTTGCATATGTACAGTCCCAGAAGTA-3’ |
|  | 132CH2_R^c9^ | 5’-TT**T**CATATGCTGCCTTTGGTTTTGGAGATGGT-3’ |
| rIgG-132-EGFP | 132Fc_F^c9^ | 5’-AAGCCTTGCATATGTACAGTCCCAGAAGTA-3’ |
|  | 132Fc_R^c9^ | 5’-TT**T**CATATGCTTTTACCAGGAGAGTGGGAGAG-3’ |

*: Superscript letters in primer name are corresponding to the steps of PCR shown in S1 Fig C and E. The detailed description is as follow:

c0: Primers used for amplification of Fd region of rFab-132-EGFP. After assembling with separately amplified EGFP without spacer, Fd-EGFP gene was inserted into pEF6/*LC-132*/IRES (S1 Fig B).

c1-c4: The same usage of c1-c4 in S2 Table.

c5: Primer for the addition of the spacer sequence at the 3’-terminus of Fd’ fragment.

c6 and c8: Primers for the elongation of the spacer sequence onto Fd’ fragment (c6) or EGFP (c8).

c7 and c9: Primers for the amplification of EGFP (c7) or Fc fragments (c9).

e: Primers for the amplification of Fd’-132-EGFP gene from pEF6/*rF(ab’)_2_-132-EGFP-2A* (S1 Fig E).

$: Under line in the sequences indicate *Xba* I or *Nde* I site. Bold “T” in 132Fc_R and 132Fc_R indicate the nucleotide substitutions for the amino acid substitution from Cys to Ser (S1 Fig C).
