# Supplementary material for: Enhancement of binding avidity by bivalent binding enables PrPSc-specific detection by anti-PrP monoclonal antibody 132
Source: PLoS One. 2019 Jun 6;14(6):e0217944. doi: 10.1371/journal.pone.0217944 (PMC6553756; doi:10.1371/journal.pone.0217944)
Supplement: S1 Text — (DOC) [file pone.0217944.s006.doc]

**Documents for supporting information**

**Supporting materials and methods**

**Cloning of heavy and light chains.**

Schematic illustration and primers for cloning of mAbs are shown in S1 Fig A and S1 Table, respectively. The cDNA encoding a part of variable regions of heavy and light chains were amplified by RT-PCR using degenerate primers reported previously . PCR products were cloned into pCRII-TOPO (Invitorgen) and nucleotide sequences were determined using a BigDye terminater v3.1 Cycle Sequencing Kit (Applied Biosystems) and ABI-3100 Avant sequencer (Applied Biosystems). After the determination of the nucleotide sequences of cDNA of the variable regions, 5’ and 3’ cDNA fragments of heavy and light chains of each mAb were amplified by 5’ and 3’ rapid amplification of cDNA end (RACE) as previously describe . Primers used for 5’ and 3’ RACE were listed in S2 Table. The cDNA fragments encoding 5’-untranslated region (UTR) and variable-region of heavy and light chains of mAb 44B1 were separately amplified by nested PCR. Then, DNA fragment coding from 5’-UTR to variable-region was assembled by assembly PCR (S1 Fig A). In the assembly PCR, two DNA fragments that have complementary region at the 5’ or 3’ terminus were assembled by denaturation and annealing process; then, assembled gene was amplified using specific primers (S1 Fig A). Full length cDNA fragments of heavy and light chains of mAbs 31C6 and 44B1 (accession numbers: HC-31C6, LC026056; LC-31C6, LC026057; HC-44B1, LC037230; LC-44B1, LC037231) were assembled into pCRII-TOPO/HC-31C6 and pCRII-TOPO/LC-31C6, and pCRII-TOPO/HC-44B1 and pCRII-TOPO/LC-44B1 respectively, as shown in S1 Fig A. Since both mAbs 132 and 31C6 are IgG_1_ and nucleotide sequence analysis showed that they shared their primary structures of constant regions, we assembled the complete light and heavy chain genes of mAb 132 (accession numbers: HC-132, LC028384; LC-132, LC028385), pCRII-TOPO/LC-132 and pCRII-TOPO/HC-132, respectively, by replacing the cDNA fragments of the variable region of heavy and light chain of pCRII-TOPO/HC-31C6 and pCRII-TOPO/LC-31C6 with the corresponding cDNA fragments of mAb 132 (S1 Fig A). For the construction of recombinant Fab gene, cDNA fragments coding VH-CH1 (Fd) region of mAbs 31C6, 44B1, and 132 were amplified from plasmids containing the corresponding heavy chain genes using specific primers, ANC3’_SLIC and 31C6Fd_R for mAb 31C6, ANC3’_SLIC and 44B1Fd_R for mAb 44B1, and ANC3’_SLIC and 132Fd_R for mAb 132, respectively. The amplified Fd fragment was subcloned into the *Xba* I site of pEF6/Myc-His in order to fuse the DNA fragment encoding Myc-His epitope at the 3’ end of Fd fragment (S1 Fig B and S2 Table). Nucleotide sequences of the cloned fragments were confirmed by DNA sequencing.

**Construction of the bicistronic expression plasmids for recombinant antibodies.**

The bicistronic expression plasmids were constructed as follows. Schematic illustration for construction is shown in S1 Fig B. The gene fragment encoding internal ribosome entry site (IRES) between the *Eco*R I and *Xba* I sites of pIRES vector (Clontech) was cloned into the the *Eco*R I and *Xba* I sites of pEF6/Myc-His to generate pEF6/IRES/Myc-His. The cDNA fragments encoding the light chain of mAbs 31C6, 44B1, and 132 that were excised from the corresponding plasmid containing light chain gene of each mAb with *Eco*R I, were cloned into the *Eco*R I site of pEF6/IRES/Myc-His. Then the cDNA fragments encoding Fd region of mAbs 31C6, 44B1, and 132 or entire heavy chain of the mAb 132 that were excised from pCRII-TOPO/Fd-31C6, pCRII-TOPO/Fd-44B1, and pCRII-TOPO/Fd-132 or pCRII-TOPO/HC-132 with *Xba* I were sequentially inserted into the *Xba* I site to make bicistronic expression plasmid for rFab-31C6, rFab-44B1, and rFab-132 or rIgG-132 (Fig 1A and S1 Fig B).

We also constructed the expression plasmid for the production of the Fab fragment and IgG using self-cleavage 2A peptide region from foot-and mouth virus (F2A) . We attempted to generate the fusion genes for rFab-132 or rIgG-132 containing F2A encoding fragment by assembly PCR (S1 Fig C without spacer and EGFP), or by replacing the heavy chain fragment with that of monovalent r132-EGFP, rF(ab’)_2_-EGFP (Fig 3A and S1 Fig D; the detail will be described later), fused with F2A. Primes for generating rFab with F2A are shown in S2 Table. For assembling the fusion gene for expressing rFab132 with F2A, DNA fragments encoding light chain without stop codon and the Fd region of mAb132 were amplified from pCRII-TOPO/LC-132 and pCRII-TOPO/Fd-132 using 132LC_F and 132LC-2A_R, and 132Fd-2A_F and 132Fd-Myc_R. F2A fragment was elongated onto both PCR products by sequentially performed PCR using primers with F2A sequences, 132LC_F and F2A_R1, and 132LC_F and F2A_R2 for light chain; 132Fd-2A_F and 132Fd_R, and F2A_F and 132Fd_R for Fd region. The assembly PCR was carried out using primers, 132LC_F and 132Fd-Myc_R (S2 Table). For assembling the fusion gene for expressing rIgG132 with F2A, the *Xba* I digested DNA fragments from pEF6/rF(ab’)_2_-132-EGFP-F2A and pEF6/IRES/rIgG-132 were once inserted into pTNT vector (Promega) to exchange the *Bam*H I digested gene fragment from pTNT/rF(ab’)_2_-132-EGFP-F2A with that from pTNT/HC-132 (S1 Fig D). After replacing the gene fragment, the entire fusion gene encoding rIgG-132-F2A excised with *Xba* I was inserted into the *Xba* I site of pEF6/Myc-His.

**Construction of the expression plasmids for r132-EGFP fosion proteins.**

To construct monovalent rFab-EGFP (Fig 3A), DNA fragments encoding Fd and EGFP were amplified from pEF6-Myc-His/HC-132 and pEGFP-C1 respectively, using ANC3’_SLIC and 132EGFP1_R, and 132EGFP1_F and EGFP_R (S3 Table). Then, entire gene encoding Fd-EGFP was assembled by assembly PCR using 132LC_F and EGFP_R (S3 Table). After cloning into pCRII-TOPO, the nucleotide sequence was determined by DNA sequencing. To construct a bicistronic expression plasmid for rFab-132-EGFP, the *Xba* I digested Fd-EGFP gene from pCRII-TOPO/Fd-132-EGFP was inserted into the pEF6/LC-132/IRES (S1 Fig B). The expression plasmid for rFab-132-EGFP fused with F2A was constructed by the same method as rIgG-132-F2A (S1 Fig D).

On the other hand, the fusion gene expressing rF(ab’)_2_-132-EGFP with F2A was assembled as shown in S1 Fig C. Primers for construction were shown in S3 Table. Mainly, each DNA fragment encoding light chain without stop codon, VH-CH1-hinge (Fd’) region, and EGFP without first methionine was amplified from pCRII-TOPO/LC-132, pCRII-TOPO/HC-132, and pEGFP-C1 using primers, 132LC_F and 132LC-2A_R for light chain, 132Fd-2A_F and Gly4Ser_R for Fd’ region, and G4S-EGFP_F1 and EGFP_R for EGFP. The F2A and/or spacer fragments were elongated onto PCR products using another set of primers, 132LC_F and F2A_R2 for light chain, F2A_F and Gly4Ser_R for Fd’ region, and G4S-EGFP_F2 and EGFP_R for EGFP. Then, fusion genes encoding Fab’-F2A and F2A-Fd’-EGFP were assembled by assembly PCR using 132LC_F and Gly4Ser_R, and F2A_F and EGFP_R, respectively. Finally, the entire fusion gene encoding rF(ab’)_2_-132-EGFP with F2A was assembled by assembly PCR using 132LC_F and EGFP_R. After TA-cloning and sequence determination, excised rF(ab’)_2_-132-EGFP-F2A gene with *Xba* I was inserted into pEF6/Myc-His.

We further assembled the fusion gene for expressing bivalent r132-EGFP, CH3 deleted rIgG-132-EGFP (rIgG(ΔCH3)-132-EGFP) and rIgG-132-EGFP (Fig 3A), with F2A. DNA fragments encoding CH2 and CH2-CH3 (Fc) regions were amplified by PCR using 132Fc_F and 132CH2_R, and 132Fc_F and 132Fc_R, respectively (S1 Fig C and S3 Table). After TA-cloning and sequence determination, each fragment excised with *Nde* I was inserted into the *Nde* I site of pEF6/rF(ab’)_2_-132-EGFP-2A. The orientation of inserted fragments was confirmed by sequencing analysis (S1 Fig C).

To construct the bicistronic expression plasmids for rF(ab’)_2_-132-EGFP, rIgG(ΔCH3)-132-EGFP and rIgG-132-EGFP, DNA fragments encoding Fd’-EGFP, Fd’-CH2-EGFP and Fd'-Fc-EGFP was amplified from corresponding expression plasmid with F2A using Fd-EGFP_F and EGFP_R. After TA-cloning and sequence determination, each *Xba* I digested fragment was inserted into the *Xba* I site of pEF6/LC-132/IRES (S1 Fig E).

**Supporting Figure legends**

**S1 Fig. Strategy for molecular cloning of antibodies and construction of the expression plasmids.** (A) Cloning of mAbs 31C6, 44B1 and 132. DNA fragments of light and heavy chains amplified by degenerate PCR, and 5’ or 3’ RACE are indicated in the same color as shown in Figs 1A and 1B. The fragments encoding variable region (VH, VL) and constant region (CH1, CL) in the amplified fragments are laid to overlap each other. The restriction enzyme in each step indicates the unique enzymes for assembling full length genes of mAbs. The left part indicates how to assemble the DNA fragments from 5’-UTR to constant region of mAb 44B1, whereas the middle part indicates the replace the VH and VL of mAb 31C6 with those of mAb 132. Names for completed plasmid are shown in bold. (B) Construction of the bicistronic plasmid with IRES. Restriction enzymes used for digestion are shown in each step. Arrows at the top and bottom on HC-132 indicate the specific primers to amplify the DNA fragment encoding Fd-132 without stop codon (S2 Table). Primers containing the *Xba* I site are indicated with purple. The expression plasmid for rFab-31C6, rFab-44B1 (Fig 1C), and rFab-132-EGFP (Fig 3A) were constructed by the same method. (C) Construction of the expression plasmid for rF(ab’)_2_-132-EGFP (Fig 3A) fused with F2A. The amplified fragments encoding light chain, Fd region and EGFP were indicated with the same color as shown in Figs 1A, 1B, and 3A. Region-specific sequences in primers (S3 Table) were drawn in dark pink (light chain), orange (F2A), red (spacer, GGGGSGGGGSGGGGS), dark green (EGFP), and black (Fd, CH2 (not shown) or Fc). The *Xba* I or *Nde* I site in primers was drawn in purple. In assembly PCR, the complementary regions in amplified fragments are laid to overlap each other. The nucleotide and deduced amino acid sequences at the connected region of rIgG-132-EGFP were shown in a square. The bold “A” and underlined letter indicates the nucleotide substitution for the mutation Cys to Ser and the *Nde* I site, respectively. (D, E) Methods for exchanging the gene fragments from (D) F2A to IRES, or (E) IRES to F2A.

**S2 Fig. Detection of PrP^C^ and anti-PrP mAbs used for crosslinking in N2a-3 cells.** N2a-3 cells were incubated with DMEM containing 10 nM IgG-31C6 (B, E, H, and K) and IgG-44B1 (C, F, I, and L) for 2 days. The cells were stained with 1 μg/ml Alexa Fluor 647-labeled mAb 132 after treatment with ((Gdn(+), G–L) or without ((Gdn(-), A–F) 5 M GdnSCN (green). Bound IgG-31C6 (E, K) and IgG-44B1 (F, L) were stained using Alexa flour 555-labeled secondary antibody against mouse Igs prior to PrP^C^ detection with Alexa Fluor 647-labeled mAb 132. Inserted image in the merged images (E, F, K, and L) are corresponding to the high-magnification image of the boxed regions. The leftmost images show negative controls for antibody-treatment (A–D). Cell nuclei were stained with DAPI (blue).

The presence of IgG-31C6 and IgG-44B1 that were used for crosslinking of the cell surface PrP^C^ was clearly demonstrated by the typical fine-dotted stains, probably on the cell membrane, with Alexa Flour 555-labeled anti-mouse Ig secondary antibody (S2 Fig E and F), which is consistent with our previous results . The IgG stains were also merged with the direct staining of Alexa Flour 647-labeled mAb 132 (S2 Fig E and F), indicating that mAb 132 reacted with the crosslinked PrP^C^. The stains of IgG-31C6 and IgG-44B1 significantly weakened after the guanidinium salt pretreatment (S2 Fig K and J). We do not know the exact reason for this. Guanidinium salt treatment that may affect the epitope for the secondary antibody on the crosslinked IgG would be one of the reasons. The detection of crosslinked PrP^C^ by mAb 132 directly labeled with Alexa Flour 647 was not affected by the guanidinium salt pretreatment (S2 Fig B vs H for IgG-31C6, C vs I for IgG-44B1).

**References**

1. Kettleborough CA, Saldanha J, Ansell KH, Bendig MM (1993) Optimization of primers for cloning libraries of mouse immunoglobulin genes using the polymerase chain reaction. Eur J Immunol 23: 206-211.

2. Horiuchi M, Ishiguro N, Nagayama K, Toyoda Y, Shinagawa M (1997) Alternative usage of exon 1 of bovine PrP mRNA. Biochem Biophys Res Commun 233: 650-654.

3. Horiuchi M, Ishiguro N, Nagayama K, Toyoda Y, Shinagawa M (1998) Genomic structure of the bovine PrP gene and complete nucleotide sequence of bovine PrP cDNA. Anim Genet 29: 37-40.

4. Stemmer W, Crameri A, Ha K, Brennan T, Heyneker H (1995) Single-step assembly of a gene and entire plasmid from large numbers of oligodeoxyribonucleotides.

5. Kim CL, Umetani A, Matsui T, Ishiguro N, Shinagawa M, Horiuchi M (2004) Antigenic characterization of an abnormal isoform of prion protein using a new diverse panel of monoclonal antibodies. Virology 320: 40-51.

6. Donnelly ML, Luke G, Mehrotra A, Li X, Hughes LE, Gani D, et al. (2001) Analysis of the aphthovirus 2A/2B polyprotein 'cleavage' mechanism indicates not a proteolytic reaction, but a novel translational effect: a putative ribosomal 'skip'. J Gen Virol 82: 1013-1025.

7. Kim CL, Karino A, Ishiguro N, Shinagawa M, Sato M, Horiuchi M (2004) Cell-surface retention of PrPC by anti-PrP antibody prevents protease-resistant PrP formation. J Gen Virol 85: 3473-3482.

8. Yamasaki T, Suzuki A, Hasebe R, Horiuchi M (2014) Comparison of the anti-prion mechanism of four different anti-prion compounds, anti-PrP monoclonal antibody 44B1, pentosan polysulfate, chlorpromazine, and U18666A, in prion-infected mouse neuroblastoma cells. PLoS One 9: e106516.
